# Nitrosylation of Nitric‐Oxide‐Sensing Regulatory Proteins Containing [4Fe‐4S] Clusters Gives Rise to Multiple Iron–Nitrosyl Complexes

**DOI:** 10.1002/anie.201607033

**Published:** 2016-10-25

**Authors:** Pauline N. Serrano, Hongxin Wang, Jason C. Crack, Christopher Prior, Matthew I. Hutchings, Andrew J. Thomson, Saeed Kamali, Yoshitaka Yoda, Jiyong Zhao, Michael Y. Hu, Ercan E. Alp, Vasily S. Oganesyan, Nick E. Le Brun, Stephen P. Cramer

**Affiliations:** ^1^ Department of ChemistryUniversity of CaliforniaDavisCA95616USA; ^2^ Physical Biosciences DivisionLawrence Berkeley National LaboratoryBerkeleyCA94720USA; ^3^ Centre for Molecular and Structural BiochemistrySchool of ChemistryUniversity of East AngliaNorwich Research ParkNorwichNR4 7TJUK; ^4^ School of Biological SciencesUniversity of East AngliaNorwichNR4 7TJUK; ^5^ University of Tennessee Space InstituteTullahomeTN37388-9700USA; ^6^ Research and Utilization DivisionSPring-8/JASRI1-1-1 Kouto, SayoHyogo679-5198Japan; ^7^ Advanced Photon SourceArgonne National LaboratoryArgonneIL60439USA

**Keywords:** gene regulation, iron–sulfur clusters, nitric oxide, nuclear vibrational resonance spectroscopy, synchrotron radiation

## Abstract

The reaction of protein‐bound iron–sulfur (Fe‐S) clusters with nitric oxide (NO) plays key roles in NO‐mediated toxicity and signaling. Elucidation of the mechanism of the reaction of NO with DNA regulatory proteins that contain Fe‐S clusters has been hampered by a lack of information about the nature of the iron‐nitrosyl products formed. Herein, we report nuclear resonance vibrational spectroscopy (NRVS) and density functional theory (DFT) calculations that identify NO reaction products in WhiD and NsrR, regulatory proteins that use a [4Fe‐4S] cluster to sense NO. This work reveals that nitrosylation yields multiple products structurally related to Roussin's Red Ester (RRE, [Fe_2_(NO)_4_(Cys)_2_]) and Roussin's Black Salt (RBS, [Fe_4_(NO)_7_S_3_]. In the latter case, the absence of ^32^S/^34^S shifts in the Fe−S region of the NRVS spectra suggest that a new species, Roussin's Black Ester (RBE), may be formed, in which one or more of the sulfide ligands is replaced by Cys thiolates.

Iron–sulfur (Fe‐S) proteins fulfill a wide range of biological functions, including electron transport, catalysis of chemical reactions, and chemical sensing.^1^ Fe‐S proteins vary in their sensitivity to NO and O_2_, but many are susceptible to damage under conditions of oxidative and nitrosative stress, resulting, for example, in deficiencies in respiration and DNA replication.^2^ The intrinsic sensitivity of the cluster is exploited through the evolution of Fe‐S proteins that function as sensors for these molecules.^3^ Reactions of NO with Fe‐S clusters usually result in the transformation of the Fe‐S core into nitrosyl iron complexes.^4^ The simplest of these are mononitrosyl iron complexes (MNICs) and dinitrosyl iron complexes (DNICs).^5^ More complex non‐homoleptic iron‐nitrosyl complexes include the multi‐iron Roussin's red ester (RRE), and Roussin's black salt (RBS; Figure 1).

**Figure 1:**
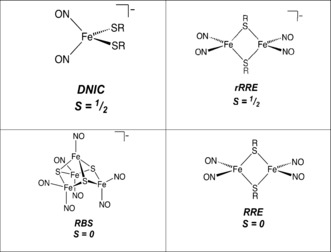
The major iron‐nitrosyl species.

Early studies of nitrosative stress using EPR spectroscopy led to the conclusion that DNIC is the principal product of Fe‐S nitrosylation, mainly because DNICs are paramagnetic (S=1/2 ) and give rise to a readily detectable signal at *g*=2.03.^6^ However, quantification of the EPR showed only a fraction of the total iron content.^7^ Other forms of spectroscopy, including FTIR, ^57^Fe Mössbauer, UV/Vis, Raman, and X‐ray absorption, have been used to study iron‐nitrosyls.^4^, ^8^ However, these techniques lack full diagnostic ability to discriminate between different iron‐nitrosyl species.

Nuclear resonance vibrational spectroscopy (NRVS)^9^ has recently been applied to iron proteins.^10^ Like IR and Raman spectroscopies, data from NRVS can be converted into ^57^Fe partial vibrational density of states (PVDOS), but the selection rules differ such that it provides a full set of the vibrational modes that involve significant displacement of the ^57^Fe nucleus along the direction of the incident X‐ray.10a, 11 Consequently, NRVS is also ^57^Fe‐specific, making it ideal for probing the intermediates and products of Fe‐S cluster nitrosylation. It was recently applied to study the reactions with NO of a [4Fe‐4S] ferredoxin^12^ and a [2Fe‐2S] Rieske‐type protein.^13^ The fragility of Fe‐S cluster proteins that function as physiological sensors of NO have so far hampered efforts to apply techniques, such as NRVS, that require millimolar concentrations and ^57^Fe‐enrichment.

The Wbl family of regulators are found only in the actinobacteria,^14^ a phylum of Gram‐positive bacteria that includes *Streptomyces* and important pathogens such as *Mycobacterium tuberculosis*. Wbl proteins are required for sporulation in *Streptomyces* and for the ability of the *M*. *tuberculosis* pathogen to persist within its host, as well as its remarkable tolerance to a wide range of antibiotics.^15^ *M. tuberculosis* WhiB3, a member of the Wbl family, is believed to function as a sensor of O_2_ and NO to control expression of genes involved in intermediary metabolism,^16^ and *M. tuberculosis* WhiB1 is known to bind DNA specifically following cluster nitrosylation,^17^ suggesting that the reaction of Wbl proteins with NO may be a common signaling process. NsrR is a widespread NO‐sensing protein that regulates the response of many bacteria to stress due to NO.^18^

NsrR and the Wbl protein WhiD (both from *S. coelicolor*) each contain a [4Fe‐4S] cluster. The former is bound by three cysteine ligands plus one oxygen ligand,^19^ and the latter by four cysteines,^20^ thus giving a comparison between the NO reactivity of two clusters with different ligations, as well as biological functions. Previous studies showed that both clusters only react slowly with O_2_, but rapidly with 8–10 NO in a multi‐phase reaction that results in protein‐bound iron‐nitrosyl species.^19^, ^20^, ^21^ EPR studies showed that DNICs are not major products. Absorbance characteristics of the iron‐nitrosyl products suggested the presence of RRE‐ or RBS‐type species, but their precise nature was not determined. A more definitive and iron‐specific method of analyzing Fe‐S protein nitrosylated products is required.

Mössbauer spectra and NRVS of WhiD and NsrR, as‐isolated, were collected before and after NO treatment. The isomer shift (δ) and quadrupole splitting (Δ*E*_Q_) parameters from the Mössbauer spectra (Figure S1, Table S1) are consistent with the presence of two pairs of high‐spin Fe^2+^/Fe^3+^ ions in each [4Fe‐4S]^2+^ cluster.^22^ Following addition of excess NO, the much lower isomer shift (δ_1_ of ≈0.16 compared to ≈0.4 mm s^−1^) and quadrupole splitting (Δ*E*_Q_ of ≈0.8 compared to >1; Figure S1, Table S1) indicate a significant change in the iron environment, consistent with the formation of iron‐nitrosyl species.^12^, ^23^ NRVS spectra are shown in Figure S2, where they are compared to the previously published NRVS spectrum of D14C variant of *Pyrococcus furiosus* [4Fe‐4S] ferrodoxin.^12^ All of the spectra are very similar, demonstrating that the [4Fe‐4S] core is intact in both WhiD and NsrR proteins. Although bands are due to collective modes involving different atomic groups, in some cases these collective motions are dominated by vibrations that are similar to local motions. Thus, by comparison with the previous work,^24^ we can assign modes and regions of the spectrum that predominantly correspond to general cluster torsion, Fe‐S bending, and Fe‐S stretching, along with the region in which N‐Fe‐N modes are observed in iron‐nitrosyl species (Figure S2). In Figure 2, the NRVS spectra for unlabeled and ^34^S‐labeled WhiD and NsrR proteins are compared. Because only the bridging S atoms are ^34^S‐labeled, the isotopic shifts further assist in normal mode identification, especially for the vibrational mode(s) related to the bridging S. The spectral region of importance, arising predominantly from Fe−S stretching modes, is 200–450 cm^−1^ (Figure S2). Being mostly collective vibrations, the magnitude of the shifts observed depends on the degree of sulfur motion in the particular vibration, meaning that isotopic substitution can result in some previously overlapping features becoming resolvable, or appearing to change in line width. For WhiD, there is a clear shift to lower energy for two major peaks of the Fe−S stretching region (ca. 240 and 390 cm^−1^) and a smaller shift at 355 nm. In NsrR, the lower energy shifts are not as evident. This may be due in part to the symmetric “breathing modes” of the protein, not present in the WhiD protein,10b which occur at a lower range of energies around 314–370 cm^−1^.^25^ However, a shift of the higher energy peak of the Fe−S stretching mode (ca. 390 cm^−1^) is observed for NsrR. These modes occur outside of the “breathing” region and therefore reflect the asymmetric nature of the Fe−S stretching mode, which are found to be 395 cm^−1^ and 385 cm^−1^ for ^32^S (unlabeled) and ^34^S‐labeled NsrR, respectively.

**Figure 2:**
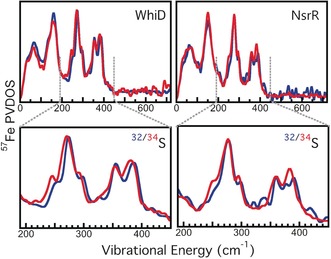
NRVS spectra of [4Fe‐4S] WhiD and NsrR. Top: ^57^Fe PVDOS for: Left (blue) unlabeled (^32^S) WhiD and (red) ^34^S‐labeled WhiD; right (blue) unlabeled (^32^S) NsrR and (red) ^34^S‐labeled NsrR. Bottom: Magnified view of the 200–450 cm^−1^ regions of the ^32/34^S WhiD (left) and NsrR (right) spectra.

Extensive changes were observed in the thiolate and N‐Fe‐N regions of the NRVS PVDOS for [4Fe‐4S] WhiD and NsrR following reaction with excess NO (Figure 3). For WhiD, the absorbance spectrum (Figure S3) confirmed that the nitrosylation product is the same as that previously observed at ≈11 NO per cluster.21a From previous studies of the reaction of NO with other [4Fe‐4S]‐containing proteins, it has been established that the Fe‐N and N‐Fe‐N regions do not overlap with Fe‐S regions.^12^ For both WhiD and NsrR, the characteristic Fe‐S peaks described above are much less intense or replaced, while there is significant new intensity in the Fe‐N‐Fe region, particularly at 530–580 cm^−1^ and 600–640 cm^−1^ (Figure 3). The peak at approximately 170 cm^−1^ for all of the spectra corresponds to Fe−S bending modes. However, the peak is significantly sharper following reaction with NO, indicating the formation of nitrosylated products with a more limited range of Fe−S bending motions than the initial Fe‐S clusters, resulting in a narrower feature observed at approximately 170 cm^−1^. The N‐Fe‐N region for ^14^NO/^15^NO‐treated WhiD and NsrR proteins (Figure 3) illustrate the shift in the Fe‐NO related peaks to lower energy upon ^15^NO labeling. For WhiD, NRVS following addition of lower levels of NO (Figure S4) revealed a transition between the spectra of Figure 2 and Figure 3.

**Figure 3:**
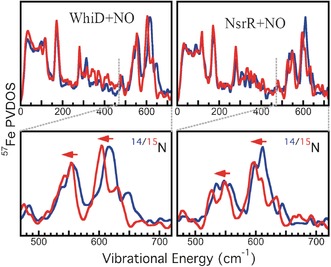
NRVS spectra of nitrosylated [4Fe‐4S] WhiD and NsrR. ^57^Fe PVDOS. Top: Left (blue) unlabeled WhiD+^14^NO; (red) unlabeled WhiD+^15^NO. Right (blue) unlabeled NsrR+^14^NO; (red) unlabeled NsrR+^15^NO. Bottom: Magnified view of N‐Fe‐N region (440 cm^−1^–740 cm^−1^).

The NRVS spectra of the two proteins contain features that are characteristic of both RRE‐ and RBS‐like species (Figure 4). For example, signals at 278, 481, and 537 cm^−1^ correspond to RBS, while features at 97, 318, and 643 cm^−1^ correspond to RRE. Furthermore, the bands in the N‐Fe‐N region of the WhiD and NsrR spectra are quite broad, consistent with the superposition of features due to RRE and RBS (Figure S5). In the case of NsrR, the RRE‐like component appears to be smaller than in WhiD. Previous EPR studies showed that there is also a minor component of DNIC species present in these samples. This is more substantial for NsrR (≈16 % total iron), consistent with the presence of features in the nitrosylated NsrR NRVS spectrum at 137, 527, and 600 cm^−1^ that correspond to DNIC signals (Figures 4 and S5B).21b

**Figure 4:**
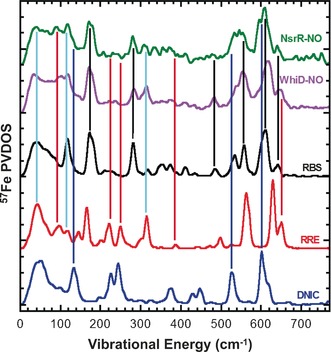
Comparison of NRVS spectra of DNIC, RRE, and RBS with those of WhiD‐NO and NsrR‐NO. Black, red, and blue lines indicate the signals that correspond to the RBS, RRE, and DNIC model compounds, respectively. Light blue lines highlight the signals that appear in both WhiD‐NO and NsrR‐NO samples and both RBS and RRE model spectra. RRE and DNIC models utilized methyl thiolate ligands.

The observation of multiple iron‐nitrosyl species accords with their chemical properties in solution, which show that an equilibrium exists between DNIC and RRE species, controlled by the availability of thiolate ligands,^26^ that Roussin's red salt (RRS, the sulfide‐bridged form of RRE) can undergo reversible conversion to RBS,^27^ and that RRS complexes can readily undergo ligand exchange with thiolate ligands.^28^ Conversion between different iron‐nitrosyl species depends on the concentration of NO, the redox states of iron and sulfur, and the availability of sulfide and thiol ligands. Our data show clearly that the Fe‐S binding pockets of both WhiD (4 Cys ligands) and NsrR (3 Cys ligands) can accommodate a variety of iron‐nitrosyl products related to those that can be generated as small molecule complexes. Previously, only the DNIC species had been identified in these two proteins.2c, 7a These results raise the question of whether the different nitrosyl forms have distinct biological functions. We showed recently that, in vitro, the effect of NO on the binding of [4Fe‐4S] NsrR to DNA is dependent on the promoter sequence.21b For the *hmpA2* promoter, only ≈2.5 NO per cluster were needed to abolish DNA binding, while for the *hmpA1* and *nsrR* promoters, ≈4 and ≈8 NO per cluster, respectively, were needed to entirely abolish binding. This implies that different iron‐nitrosyl species are indeed involved in activating/deactivating DNA binding.

[4Fe‐4S] WhiD and NsrR labeled with ^34^S sulfides were also reacted with NO, and NRVS spectra were recorded (Figures S6 and S7). Unlike the ^32^S/^34^S [4Fe‐4S] spectra, no clear shifts were observed in the spectra of the nitrosylated samples compared to NO‐treated natural abundance (^32^S) samples. This is a key observation because it shows that there is no significant amount of ^34^S (that is, bridging S) bound directly to Fe in the nitrosylated forms, ruling out RBS itself as a product of the nitrosylation.

To assist with spectral assignments, and in particular to determine the effects of isotopic substitution (^34^S and ^15^N), and to investigate the possibility of persulfide formation and coordination of iron‐nitrosyls, we performed a normal mode analysis using density functional theory (DFT) calculations. Similar calculations were previously successfully carried out for a [4Fe‐4S] cluster.^24^ Calculations for RRE and RBS iron‐nitrosyl species are shown in Figures S8 and S9. In general, the lower energy region of the spectra consists of peaks formed from a combination of different vibrations. The mid‐energy region is mostly S‐Fe‐S vibrations, and the upper energy region is mostly strong N‐Fe‐N motions. The RRE calculations (Figure S8) show good agreement with the previously reported ^57^Fe PVDOS of [Fe_2_(μ‐SPh)_2_(NO)_4_].^12^ Calculations with ^15^N in place of ^14^N showed that bands in the 500–650 cm^−1^ region are sensitive to ^14/15^N substitution, as found experimentally for WhiD and NsrR. The RBS calculations (Figure S9) also show good agreement with the previously reported ^57^Fe PVDOS of (Et_4_N)[Fe_4_(μ_3_‐S)_3_(NO)_7_],^12^ particularly in terms of the pattern of bands and their relative intensities right across the spectrum. Calculations with ^15^N in place of ^14^N and ^34^S in place of sulfide ^32^S (with Cys thiolate sulfur remaining as ^32^S) revealed that bands in the 500–650 cm^−1^ region are sensitive to ^14/15^N substitution, and bands in the 240–380 cm^−1^ region are sensitive to ^32/34^S (sulfide) substitution. While ^14/15^N shifts were observed experimentally for the products of the NO reactions of WhiD and NsrR, ^32/34^S shifts were not, providing further support for the conclusion that RBS is not a nitrosylation product. Indeed, because all of the coordination positions at the irons are satisfied by non‐protein ligands (NO and bridging sulfides) the RBS ion can only be bound to a protein by ionic forces and/or hydrogen bonding. Therefore, we considered whether Roussin's black ester (RBE) species (Figures S10–S12), having one, two, or three thiolate groups (from protein Cys residues) in place of bridging sulfides, might be present. DFT calculations of the ^57^Fe PVDOS for each of these putative complexes revealed only relatively minor changes owing to these substitutions (Figures S10–S12), and a diminishing ^32/34^S shift. Therefore, on the basis of the DFT calculations, RBS‐ and RBE‐type species, particularly those with only one or two thiolate bridges, are not readily distinguishable by NRVS, and so the data are not inconsistent with the presence of RBE‐type species.

Nitrosylation of [4Fe‐4S] WhiD was previously shown to result in oxidation of sulfide to S^0^.21a This has also been reported in studies of model complexes.23a For WhiD, at least some of the S^0^ is retained by the protein in the form of Cys persulfide, raising the possibility that iron‐nitrosyl species formed might have coordination by Cys persulfides in addition to Cys thiolates.^29^ DFT calculations of the ^57^Fe PVDOS for a persulfide form of the mono‐thiol RBE were performed (Figure S13). There are relatively few differences between this and the calculated spectrum for the mono‐thiol RBE (Figure S10), and indeed that of RBS itself (Figure S7). Similar calculations were performed for single and double persulfide forms of RRE (Figures S14 and S15). While the calculated spectrum of the single persulfide form is very similar to that of RRE, that of the double persulfide form has some significant differences, particularly in the 100–200 and >600 cm^−1^ regions, suggesting that this species is distinguishable from RRE and a single persulfide adduct.

In summary, the combination of ^57^Fe Mossbauer, NRVS, and DFT, along with ^14^NO/^15^NO and ^32^S/^34^S (sulfide) substitutions have revealed that nitrosylation of the NO‐sensing [4Fe‐4S] cluster‐containing regulatory proteins WhiD and NsrR gives rise to a range of products, including variable but small (≤16 %) amounts of DNIC, but principally to species related to RBS and RRE. The former are not RBS itself because no ^32/34^S sulfide shifts were observed in the NRVS spectra. To explain these findings, we suggest the formation of a new Black Salt in which one or more of the sulfides are substituted by a Cys thiolate, resulting in a so‐called Roussins’ Black Ester (RBE). We have also uncovered evidence of Cys persulfides that may ligate the cores of RRE and RBE in these proteins. We await the preparation of inorganic model compounds that will help further in characterizing these protein‐bound forms of iron‐nitrosyls.

## Supporting information

As a service to our authors and readers, this journal provides supporting information supplied by the authors. Such materials are peer reviewed and may be re‐organized for online delivery, but are not copy‐edited or typeset. Technical support issues arising from supporting information (other than missing files) should be addressed to the authors.

Supplementary
