# Supplementary material for: Nitrosylation of Nitric‐Oxide‐Sensing Regulatory Proteins Containing [4Fe‐4S] Clusters Gives Rise to Multiple Iron–Nitrosyl Complexes
Source: Angew Chem Int Ed Engl. 2016 Oct 25;55(47):14575–9. doi: 10.1002/anie.201607033 (PMC5204455; doi:10.1002/anie.201607033)
Supplement: Supplementary file 1 — Supplementary [file ANIE-55-14575-s001.pdf]

Angewandte Chemie
Eine Zeitschrift der Gesellschaft Deutscher Chemiker

Supporting Information

Nitrosylation of Nitric-Oxide-Sensing Regulatory Proteins Containing [4Fe-4S] Clusters Gives Rise to Multiple Iron-Nitrosyl Complexes

Pauline N. Serrano, Hongxin Wang, Jason C. Crack, Christopher Prior, Matthew I. Hutchings, Andrew J. Thomson, Saeed Kamali, Yoshitaka Yoda, Jiyong Zhao, Michael Y. Hu, Ercan E. Alp, Vasily S. Oganesyan, Nick E. Le Brun, and Stephen P. Cramer**

anie_201607033_sm_miscellaneous_information.pdf

Table of Contents **Page**

**Experimental section**	3
Figure S1. Mössbauer spectra of Fe-S cluster regulatory protein before and after reaction with NO.	6
Table S1. Summary of refined Mössbauer parameters for WhiD and NsrR before and after nitrosylation	7
Figure S2. NRVS spectra of WhiD and NsrR	8
Figure S3. WhiD nitrosylation followed by absorbance spectroscopy	9
Figure S4. NRVS analysis of WhiD as a function of NO concentration	10
Figure S5. Overlay of WhiD and NsrR iron nitrosyl NRVS spectra with those of RRE, RBS and DNIC complexes	11
Figure S6. Overview of NRVS spectra for nitrosylated WhiD	12
Figure S7. Overview of NRVS spectra for nitrosylated NrsR	13
Figure S8. DFT calculated NRVS spectra of RRE	14
Table S2. DFT calculated vibrational modes and frequencies for RRE	15
Figure S9. DFT calculated NRVS spectra of RBS	16
Table S3. DFT calculated vibrational modes and frequencies for RBS	17
Figure S10. DFT calculated NRVS spectra of RBE with one thiolate bridge	18
Table S4. DFT calculated vibrational modes and frequencies for RBE with one thiolate bridge	19
Figure S11. DFT calculated NRVS spectra of RBE with two thiolate bridges	20
Table S5. DFT calculated vibrational modes and frequencies for RBE with two thiolate bridges	21
Figure S12. DFT calculated NRVS spectra of RBE with three thiolate bridges	22
Table S6. DFT calculated vibrational modes and frequencies for RBE with three thiolate bridges	23
Figure S13. DFT calculated NRVS spectra of RBE with one persulfide thiolate bridge	24
Table S7. DFT calculated vibrational modes and frequencies for RBE with one persulfide thiolate bridge	25
Figure S14. DFT calculated NRVS spectra of RRE with one persulfide bridge	26
Table S8. DFT calculated vibrational modes and frequencies for RRE with one persulfide bridge	27
Figure S15. DFT calculated NRVS spectra of RRE with two persulfide bridges	28
Table S9. DFT calculated vibrational modes and frequencies for RRE with two persulfide bridges	29
**Supporting References**	30

Experimental Section

Preparation of $^{57}Fe/^{34}S$-enriched [4Fe-4S] NsrR. Soluble [4Fe-4S]-NsrR (holo-NsrR) was over produced from plasmid pNsrR in aerobic *E. coli* cultures (BL21 λDE3 Star, Novagen; 37 °C), purified, and converted to apo-NsrR as previously described.[1] Isotopically enriched [4Fe-4S]-NsrR was prepared via a standard NifS catalysed *in vitro* reconstitution (50 mM Tris, 50 mM NaCl, 5% (v/v) glycerol, pH 8.0) in the presence of $^{57}FeCl_3$, and ^{34}S-cysteine, as previously described.[1b, 2] Isotopically enriched NsrR was isolated and concentrated via a HiTrap heparin column and eluted in 50 mM Tris 2 M NaCl, 5% (v/v) glycerol pH 8.0, as previously described.[1b] Holo-NsrR with a ^{57}Fe enriched [4Fe-4S] cluster was obtained by supplementing the growth medium with $^{57}FeCl_3$ (Goss Scientific Ltd).[1b] The CD spectrum of the isotopically enriched samples were identical to that of natural abundance samples (not shown).[1c] Where necessary, a centrifugal spin concentrator (Amicon Ultra, 10K MWCO, regenerated cellulose) was used to concentrate the sample.

Preparation of $^{57}Fe/^{34}S$-enriched [4Fe-4S] WhiD. Soluble ^{57}Fe enriched [4Fe-4S]-WhiD (holo-WhiD) was over produced from plasmid pIJ6631 as a $(His)_6$-tagged protein in aerobic *E. coli* cultures (BL21 λDE3 Star, Novagen; 37 °C), supplemented with $^{57}FeCl_3$ (Goss Scientific Ltd). Holo-WhiD was purified on a HisTrap column, as previously described,[3] and buffer exchanged into 25 mM HEPES, 2.5 mM $CaCl_2$, 100 mM NaCl, 100 mM $NaNO_3$, pH 7.5 via a PD10 column (GE Life Sciences). Incorporation of [^{34}S] sulfide (Goss Scientific Ltd) into the cluster was carried out via sulfide exchange, as described by Kennedy *et al*.[4] Briefly, [^{34}S] sulfide (4.15 mM final conc.) and DTT (2.5 mM) were added to holo-WhiD (28 µM [4Fe-4S]) and incubated at ~46 °C for 3 hr. The sample was then rapidly cooled to ~25 °C, treated with a further aliquot of DTT (to give 4.9 mM final conc.), passed through a 0.2 µm filter, and buffer exchanged into 50 mM Tris, 800 mM NaCl, 5% (v/v) glycerol, pH 7.3. Where necessary, a centrifugal spin concentrator (Amicon Ultra, 10 K MWCO, regenerated cellulose) was used to concentrate the sample. Control $^{32}S/^{34}S$ exchange experiments with WhiD containing natural abundance ^{56}Fe confirmed specific enrichment of the [4Fe-4S] cluster with the [^{34}S] sulfide isotope. The CD spectra of the isotopically enriched samples were identical to non-enriched samples (not shown).[3]

Electrospray ionization mass spectroscopy under non-denaturing conditions[1c] was used to confirm specific isotope enrichment of [4Fe-4S] WhiD. Briefly, holo-WhiD was exchanged into 250 mM ammonium acetate pH 7.1 using a Zeba spin desalting column (Thermo scientific), diluted to ~6 µM cluster (6 pmol/µl), and infused directly (0.3ml/hr) into the ESI source of a Bruker micrOTOF-QIII mass spectrometer (Bruker, Coventry, UK) operating in the positive ion mode. Full mass spectra (m/z 50 – 3500) were recorded for 5 min. Spectra were combined, processed using the ESI Compass 1.3 Maximum Entropy deconvolution routine in Bruker Compass Data analysis 4.1 (Bruker Daltonik GmbH). The mass spectrometer was calibrated with ESI-L low concentration tuning mix in the positive ion mode (Agilent Technologies). Masses below are given as the isotope average of the neutral protein or protein-cluster complex. A peak corresponding to apo-WhiD was observed at a mass of 14025 Da. The predicted mass was 14159 Da indicating the loss of the N-terminal Met residue (14159 – 131 = 14208 Da) and the likely oxidation of Cys thiols to form a disulfide (14208 -2 = 14026 Da). Non-enriched holo-WhiD samples had an observed mass of 14377 Da (protein +352 = 14377 Da). Samples containing either ^{57}Fe, or ^{34}S, had an observed mass of 14381 Da (protein +356 = 14381 Da) and 14384 Da (protein +360 = 14385 Da), respectively. The doubly (^{57}Fe and ^{34}S) enriched sample had an observed mass of 14389 Da (protein +364 = 14389 Da). The observed mass shifts were +4, +7 and +12 Da for ^{57}Fe, ^{34}S, and $^{57}Fe/^{34}S$, respectively, consistent with full, or near-full, isotope enrichment.

Preparation of nitrosylated [4Fe-4S] NsrR and WhiD. Nitric oxide has a solubility limit of ~2 mM (at 20 °C), therefore ProliNONOate, a fast ($t_{½}$ ~2 s, Caymen Chemicals) releasing nitric oxide donor, was chosen as a convenient method of rapidly delivering excess NO to the sample. Briefly, a 50 µl aliquot of holo-protein (NsrR or WhiD), containing a minimum of ~1 mM [4Fe-4S], was combined with varying amounts of ~300 mM ProliNONOate (dissolved in 50 mM NaOH) in a 0.2 ml microtube. The solubility properties of NO limited the ratio of NO to cluster at any one time to < 2:1, but with sufficient NO released over time to achieve the desired overall stoichiometry, which in most cases was an excess (theoretical maximum of 70 NO per cluster but this does not take into account loss of gas to headspace) sufficient to drive the reaction to completion. Samples were allowed to react at ambient anaerobic box temperature (~20 °C) for 5 min (approximately 100 half-lives). Absorbance measurements of a 40-fold diluted sample of nitrosylated WhiD NRVS samples confirmed that it was identical to that

previously obtained by titration at lower concentration.[1c] Samples were loaded into NRVS cuvettes and frozen in liquid nitrogen. For samples containing ^{15}N-nitric oxide, 50 µl of holo-protein was placed in a 0.5 ml microtube and the headspace flushed with 1 ml of ^{15}N-nitric oxide gas (CIL, CKGas) and the sample gently agitated at ambient temperature for 5 min. The headspace of the sample was replaced a further two times before the sample was loaded into an NRVS cuvette and frozen. The solubility properties of NO limited the total ratio of NO to original cluster in solution to ~10, with excess NO remaining in the headspace to ensure reaction went to completion. Samples prepared in the absence of nitric oxide donors or gas served as a control.

NRVS Measurements. Samples of ~1 mM [4Fe-4S] ^{57}Fe-enriched WhiD ($^{32/34}$S labeled), NsrR ($^{32/34}$S labeled), WhiD + $^{14/15}$NO, NsrR + $^{14/15}$NO were loaded into 3 × 10 × 1 mm^3 (interior dimensions) Lucite sample cells encased with Kapton tape and frozen in liquid nitrogen. ^{57}Fe NRVS spectra were recorded using published procedures[5] at 03-ID at the Advanced Photon Source and at BL09XU at Spring-8.[6] Flux was on the order of ~ 1.4 x 10^9 photons/s in a ~ 0.8 meV bandwidth at 14.4125 keV in a 0.6 mm (vertical) x 1 mm (horizontal) spot. Delayed nuclear fluorescence and delayed Fe K fluorescence (from internal conversion) were recorded with a single 1-cm^2 square avalanche photodiode (APD) (at APS) or a 2×2 APD array (at Spring-8). Each scan took about 45 minutes and all scans were normalized to the intensity of the incident beam and then averaged according to their cts/s signal level. Partial vibrational densities of states (PVDOS) were calculated from the raw NRVS spectra using the PHOENIX software package.[7] During data collection the sample was maintained at low temperature using a liquid He cryostat (head temperature <10 K). Accurate sample temperatures were calculated from the ratio of the anti-Stokes to Stokes intensity by the expression $S(-E) = S(E)e^{-E/kT}$ and were 50-80 K.[8]

Mössbauer Measurements. Measurements were performed using a MS4 spectrometer operating in the constant acceleration mode in transmission geometry, and at 10 K for all samples using a Janis SVT-400 cryostat. A 100 mCi ^{57}Co in Rh held at room temperature was used as source. All centroid shifts, δ, are given with respect to metallic-Fe at room temperature. The spectra were least square fitted using Recoil software.[9] The parameters from these fittings are centroid shifts (δ), quadrupole splitting (ΔE_Q), Lorenztian linewidth (Γ), and intensity (*I*). During the fitting procedure, all parameters were set to be free, for which the two signals were each locked to 50% intensity. This was done because each of these doublets is related to two identical Fe atoms in the cluster. However, even when the intensities were not locked, the hyperfine values were virtually unchanged. The discrepancy in the intensity ratios of the two doublets are likely due to recoil-free fractions of different Fe sites.

DFT Calculations. All calculations were performed using the Gaussian 09 set of programs in the gas phase employing the PW91 functional.[10] Iron atoms were described using the LAN2LDZ basis set and effective core potential, with all other atoms being described with the all electron 6-311G** basis set as used by Mitra *et al*.[11] All structures were optimized and confirmed as minima during the frequency analysis. NRVS spectra were predicted from the DFT outputs using the approximation of Einstein-like modes, where there is no momentum dependence of vibrational frequency and polarization vector, and under the condition of random distribution of molecules.[5a, 12] Calculation of NRVS using harmonic DFT frequencies is a well established approach demonstrating a relatively accurate reproduction of experimental spectral features including isotopic shifts.[6e, 11, 13] As has been demonstrated previously[14] the effects of anharmonicity have much smaller impact on the difference between the experimental and calculated NRVS spectra compared to the error intrinsic to the chosen DFT/basis set combination.

The partial density of states, PVDOS, was calculated according to the following equation.[5a, 12a-h]

$$D_{Fe}(\nu) = \frac{1}{3}\sum_{\alpha} e^2_{Fe,\alpha} \cdot f(\nu - \nu_{\alpha}) \quad (1)$$

where composition factor, $e^2_{Fe,\alpha}$, for the resonant atom *Fe* in the normal mode *α* is given by:

$$e_{Fe,\alpha}^2 = \frac{\left(\Delta r_{Fe,\alpha}\right)^2 m_{Fe}}{\sum_{k=1}^{N}\left(\Delta r_{k,\alpha}\right)^2 m_k} \qquad (2)$$

In the latter expression $(\Delta r_{k,\alpha})^2$ is the mean square displacement of atom k in mode α and summation in the denominator is taken over all atoms; m_k is the mass of the kth atom. N defines the total number of atoms and $f(\nu-\nu_\alpha)$ is the line shape function which is a convolution of Gaussian and Lorentzian forms. The extracted normal modes were used to simulate the NRVS spectra according to equation (1) using an in-house built computer program.[12i] Calculated spectral lines were assigned a Lorentzian line shape with the line width of 6 cm^{-1} for PVDOS.

Supporting Figures and Tables

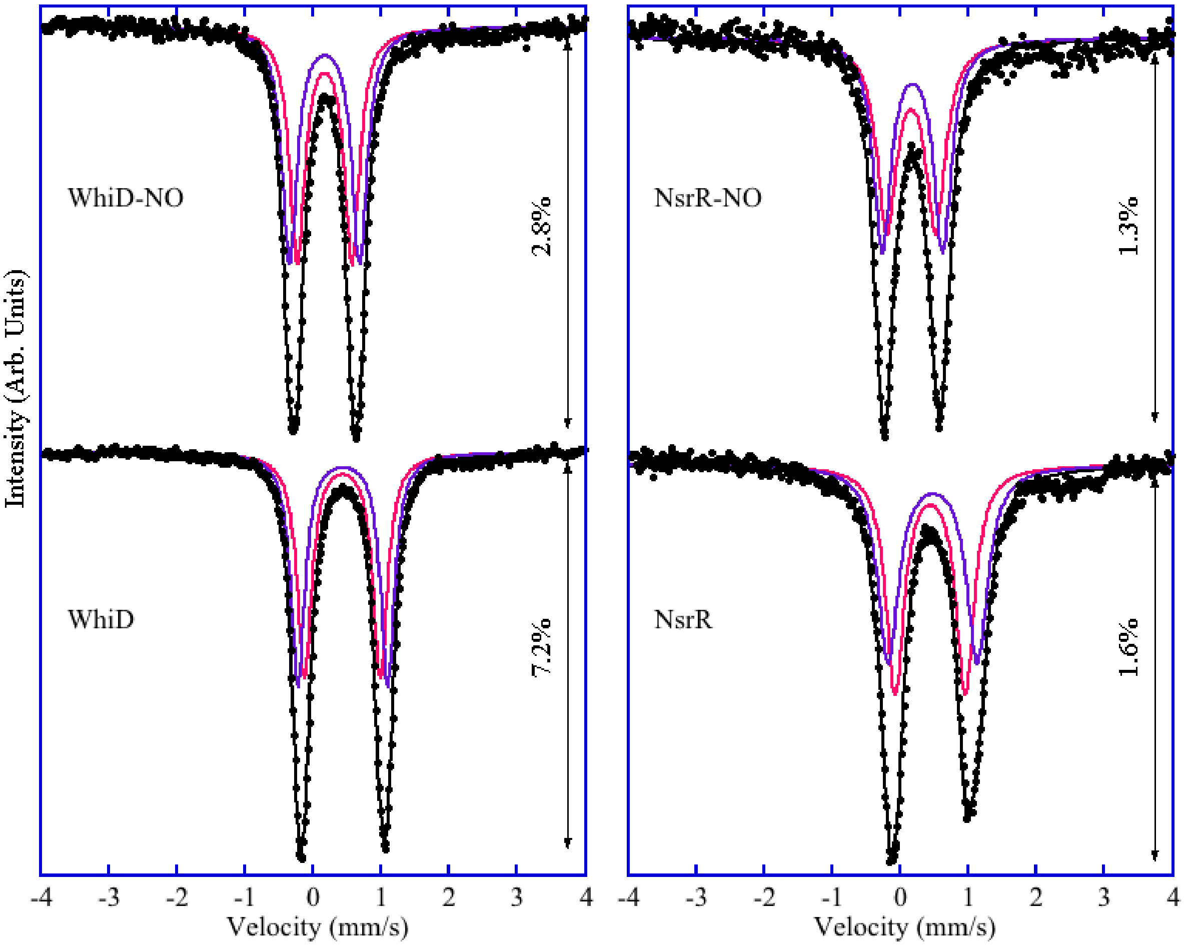

Figure S1. **Mössbauer spectra of Fe-S cluster regulatory protein before and after reaction with NO.** A) and B) Zero field Mössbauer spectra for WhiD and NsrR, respectively, as isolated and after addition of excess NO at 10 K. Due to a small asymmetry in the signals, the spectra of the two proteins were both fitted with two doublets, resulting in similar values in δ and ΔE_Q (see Table S2), indicated that two pairs of high spin Fe^{2+}/Fe^{3+} ions exist in each $[4Fe\text{-}4S]^{2+}$ cluster. Here, a delocalized electron oscillates between the ions giving rise to an average oxidation state of $Fe^{2.5+}$ for each pair, where the δ value is the average of the two δ values for high-spin Fe^{2+} and Fe^{3+}. These results are also similar to those recently reported for [4Fe-4S] NsrR.[1c] Following addition of excess NO, the Mössbauer spectra of WhiD (A) and NsrR (B) were still fitted with two similar doublets with δ and ΔE_Q parameters similar to each other but very different from those derived from the initial $[4Fe\text{-}4S]^{2+}$ spectra (see Table S1). Previous DFT calculations indicated that DNIC species are best described by two resonance structures consisting of high spin Fe^{3+} bound to two NO^- ligands and high spin Fe^{2+} bound to an overall quartet $^4(NO)_2^-$ ligand, with antiferromagnetic coupling such that a total spin of S = ½ results.[15]

Table S2. Summary of refined Mössbauer parameters for WhiD and NsrR before and after nitrosylation: Isomer shift, δ; quadrupole splitting, ΔE_Q, Lorenztian linewidth (Γ), and intensity (*I*).

**Components**	**WhiD**	**WhiD-NO**	**NsrR**	**NsrR-NO**
**δ_1 (mm/s)**	0.435	0.174	0.442	0.163
**ΔE_{Q1} (mm/s)**	1.113	0.801	1.031	0.718
**Γ_1 (mm/s)**	0.26	0.27	0.33	0.35
**I_1 (%)**	50	50	50	50
**δ_2 (mm/s)**	0.441	0.173	0.481	0.181
**ΔE_{Q2} (mm/s)**	1.317	1.029	1.309	0.887
**Γ_2 (mm/s)**	0.25	0.27	0.38	0.31
**I_2 (%)**	50	50	50	50

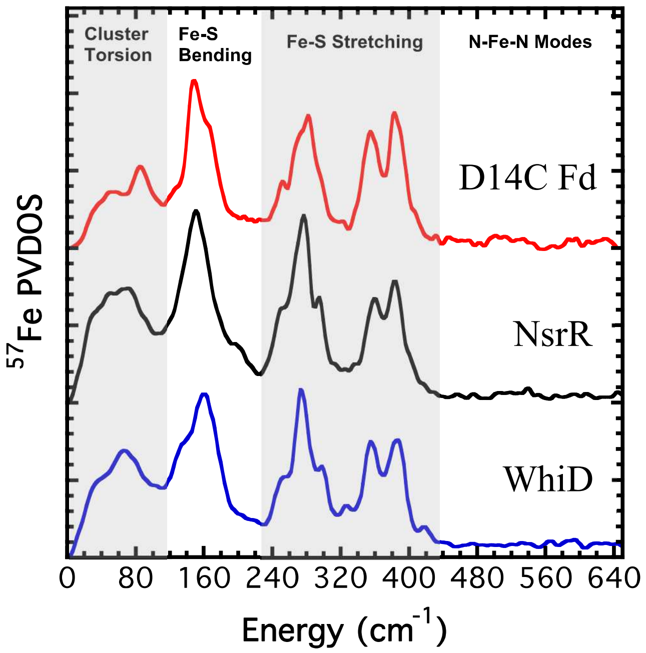

***Figure S2*. NRVS spectra of WhiD and NsrR.** ^{57}Fe PVDOS of: (bottom) [4Fe-4S] WhiD; (middle) [4Fe-4S] NsrR; (top) oxidized ferrodoxin (D14C Fd).[16]

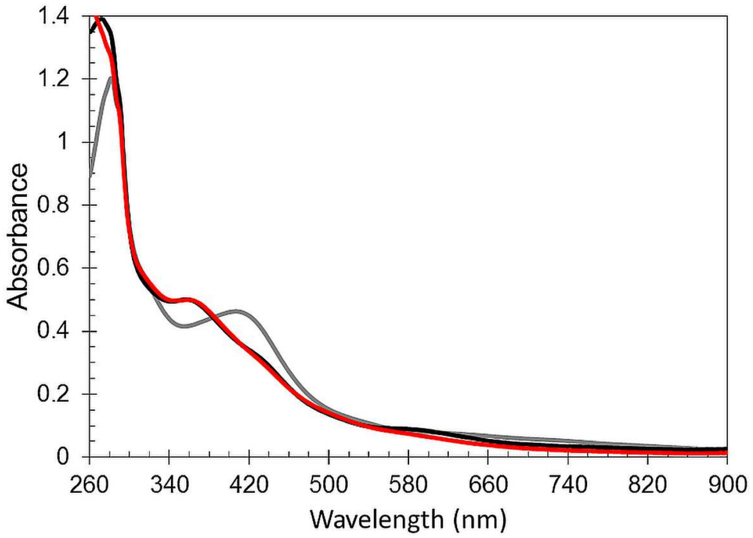

***Figure S3*. WhiD nitrosylation followed by absorbance spectroscopy.** UV-visible absorbance spectra of as isolated WhiD (grey) and following addition of ~11 NO per [4Fe-4S] cluster (black, previously reported[17]), and the nitrosylated WhiD NRVS sample following ~40-fold dilution (red).

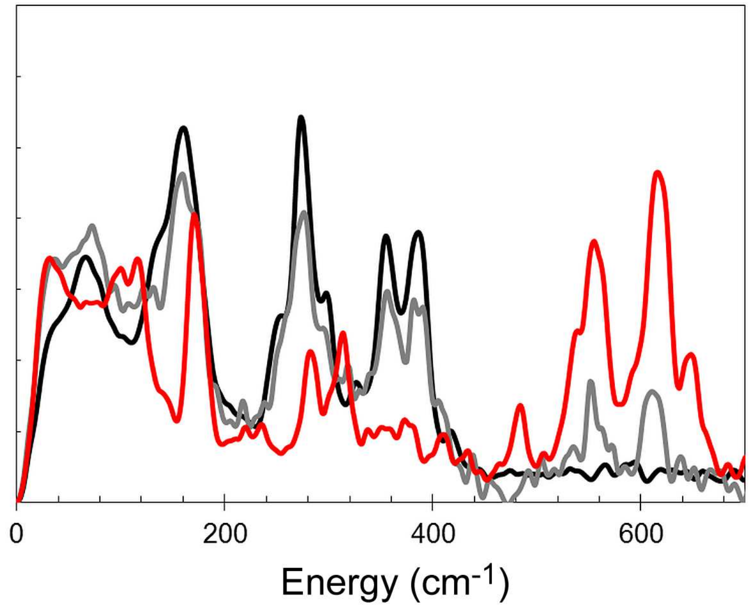

***Figure S4*. NRVS analysis of WhiD as a function of NO concentration**. NRVS spectra for as isolated [4Fe-4S] WhiD (black) and following addition of 5 NO per cluster (grey) and excess NO (up to a maximum of 48 per cluster, red).

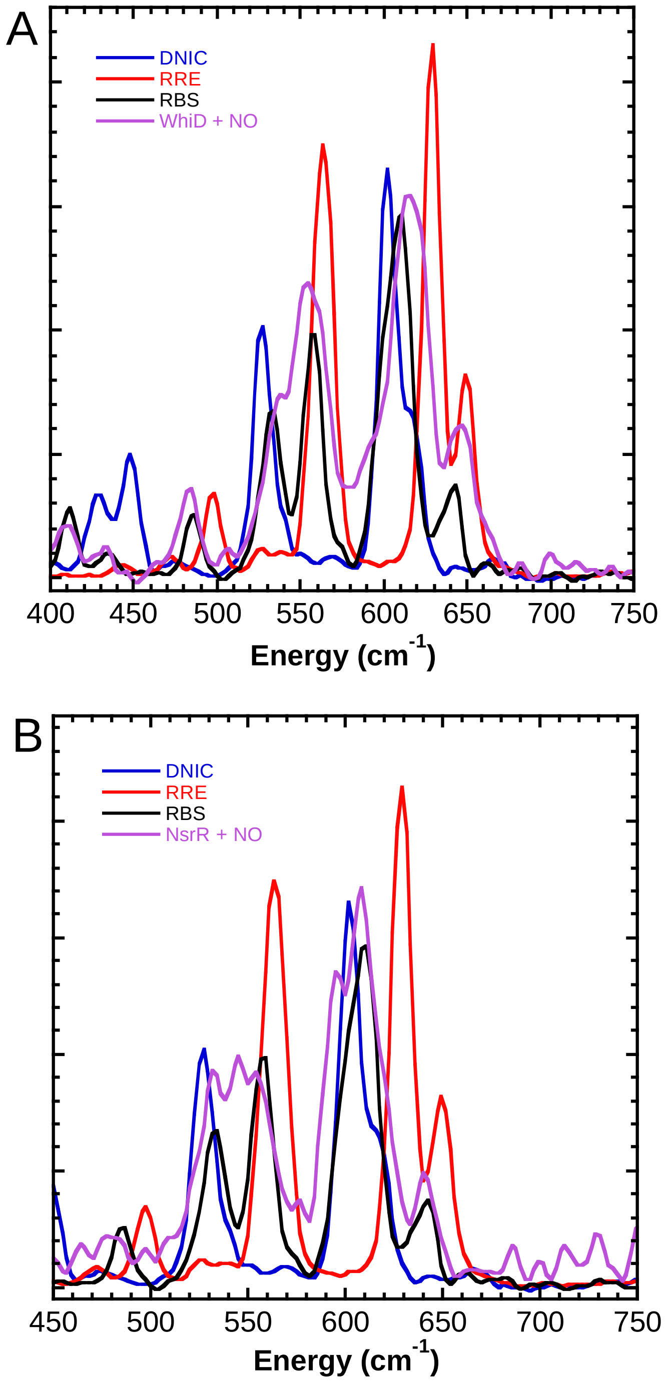

***Figure S5*. Overlay of WhiD and NsrR iron nitrosyl NRVS spectra with those of RRE, RBS and DNIC complexes.** A) WhiD, and B) NsrR.

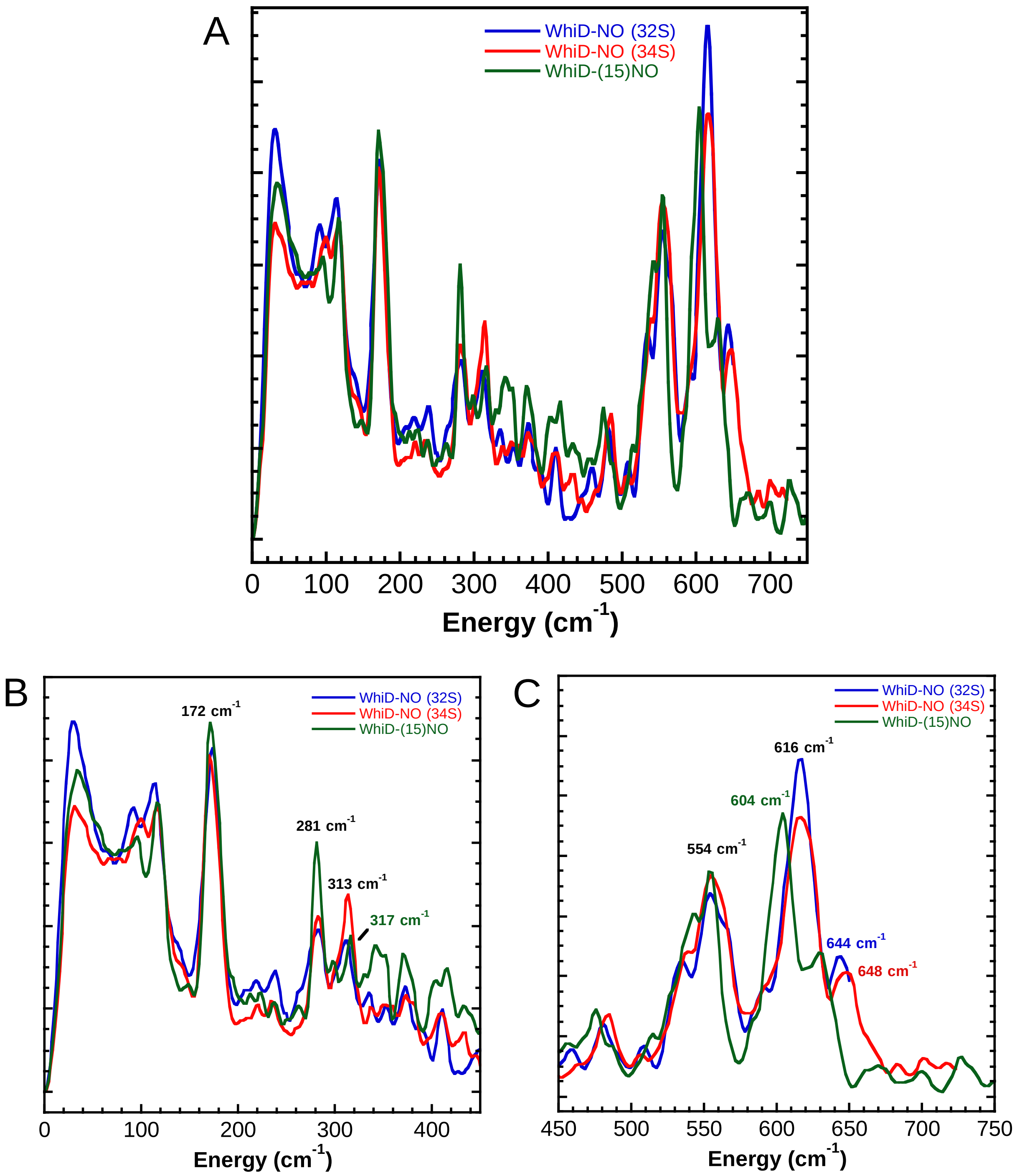

***Figure S6*. Overview of NRVS spectra for nitrosylated WhiD.** A) NRVS Spectra for $^{32}S/^{14}NO$, $^{34}S/^{14}NO$ and $^{32}S/^{15}NO$ labeled [4Fe-4S] WhiD following addition of excess NO, as indicated. B) and C) show the Fe-S and Fe-N regions in more detail.

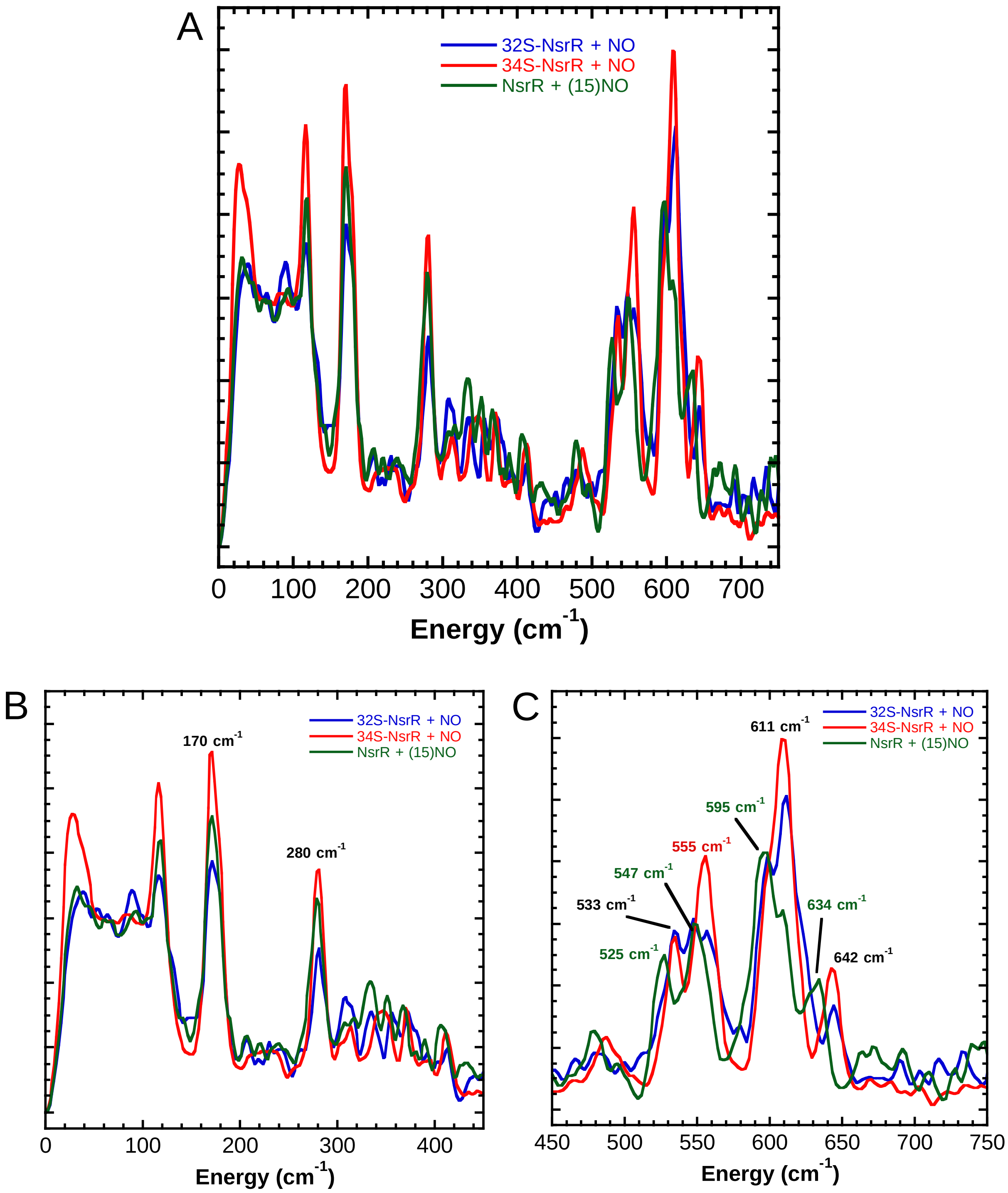

***Figure S7*. Overview of NRVS spectra for nitrosylated NrsR**. A) NRVS Spectra for $^{32}S/^{14}NO$, $^{34}S/^{14}NO$ and $^{32}S/^{15}NO$ labeled [4Fe-4S] NsrR following addition of excess NO, as indicated. B) and C) show the Fe-S and Fe-N regions in more detail.

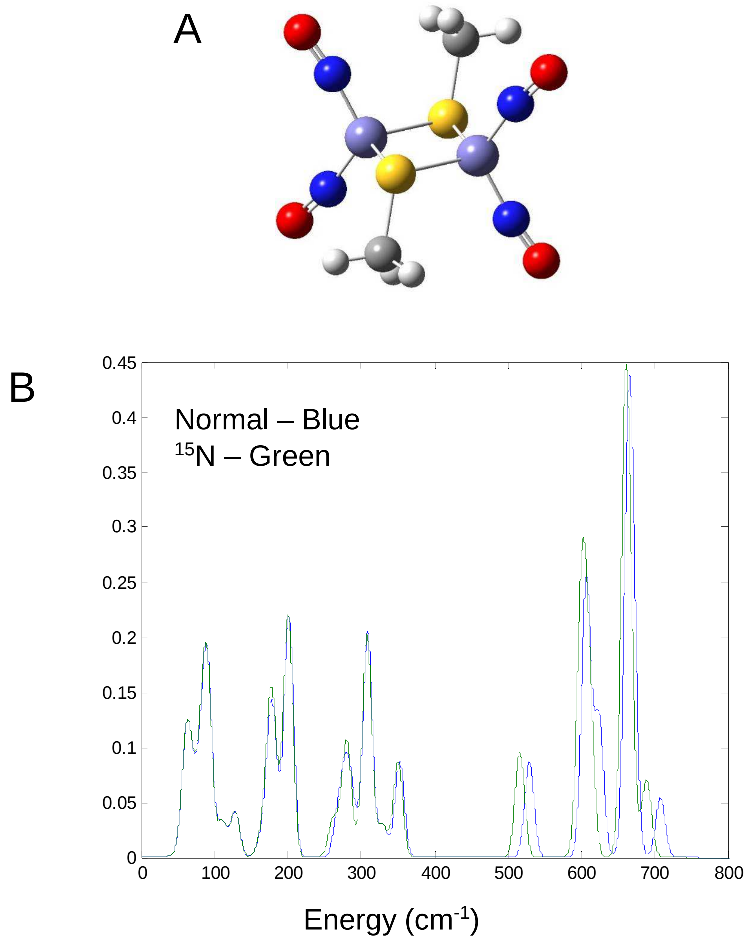

***Figure S8*. DFT calculated NRVS spectra of RRE.** A) Structure of methyl thiolate form of RRE, $[Fe_2(\mu\text{-}SCH_3)_2(NO)_4]$ (C_{2h}). Iron atoms are in light blue, sulfur in yellow, nitrogen in dark blue, oxygen in red, methyl groups in gray. B) DFT calculated NRVS spectra of RRE and ^{15}NO-subsituted RRE, as indicated. The simulation shows good agreement with the previously reported ^{57}Fe PVDOS of $[Fe_2(\mu\text{-}SPh)_2(NO)_4]$.[16] Agreement is less good above 500 cm^{-1}, but the pattern and relative intensities of bands observed are well reproduced. The broad feature below 100 cm^{-1} results from a combination of S-Fe-S twists and wags with slight N-Fe-N motion. The first sharp feature at ca. 175 cm^{-1} results from S-Fe-S twisting/scissoring with some S-Me motion as a result. The band at ca. 201 cm^{-1} is principally S-Fe-S scissor/stretching, while those at ca. 280 and 310 cm^{-1} are both due to S-Fe-S asymmetric stretches with some N-Fe-N recoil. The mid intensity feature at ca. 530 cm^{-1} results from N-Fe-N wagging and the peaks at ca. 605 cm^{-1} and 665 cm^{-1} are from N-Fe-N symmetric and asymmetric stretching modes, respectively. Calculations were also performed with ^{15}N in place of ^{14}N. Consistent with our assignments, bands in the 500-650 cm^{-1} region are sensitive to $^{14/15}N$ substitution, as also found experimentally for WhiD and NsrR.

***Table S2*. DFT calculated vibrational modes and frequencies for methyl thiolate form of RRE, [Fe_2(μ-SCH_3)$_2$(NO)$_4$].** The highlight colors indicate vibrations that are: strong in NRVS and Raman (green); strong in NRVS and IR (red).

**Mode**	**DFT Frequency (cm^{-1})**
S-Fe-S Wag	80.99
O-N-Fe-N-O Rock / Fe-S-Fe Twist	90.49
S-Fe-S Twist / Me-S-Fe Bend	175.72
Fe-S-Fe Twist / Me-S-S-Me Twist	181.06
S-Fe-S Twist / Fe-S-Fe Scissor / S-S-Me Bend	201.39
S-Fe-S Scissor/Stretch	279.30
N-Fe-N Rock / O-N-Fe Bend / S-Fe-S/Fe-S-Fe Asymmetric Stretch	309.00
N-Fe-N Rock / O-N-Fe Bend / S-Fe-S Asymmetric Stretch	352.68
N-Fe-N Wags	529.68
N-Fe-N Symmetric Stretches / Fe-S-Fe Rock	606.60
N-Fe-N Symmetric Stretches / Fe-S-Fe Scissor	608.09
N-Fe-N Wag / Fe-S-Fe Symmetric Stretch	624.81
N-Fe-N Asymmetric Stretch / Fe-S-Fe Twist	665.34
N-Fe-N Asymmetric Stretch / Fe-S-Fe Wag	666.99

A

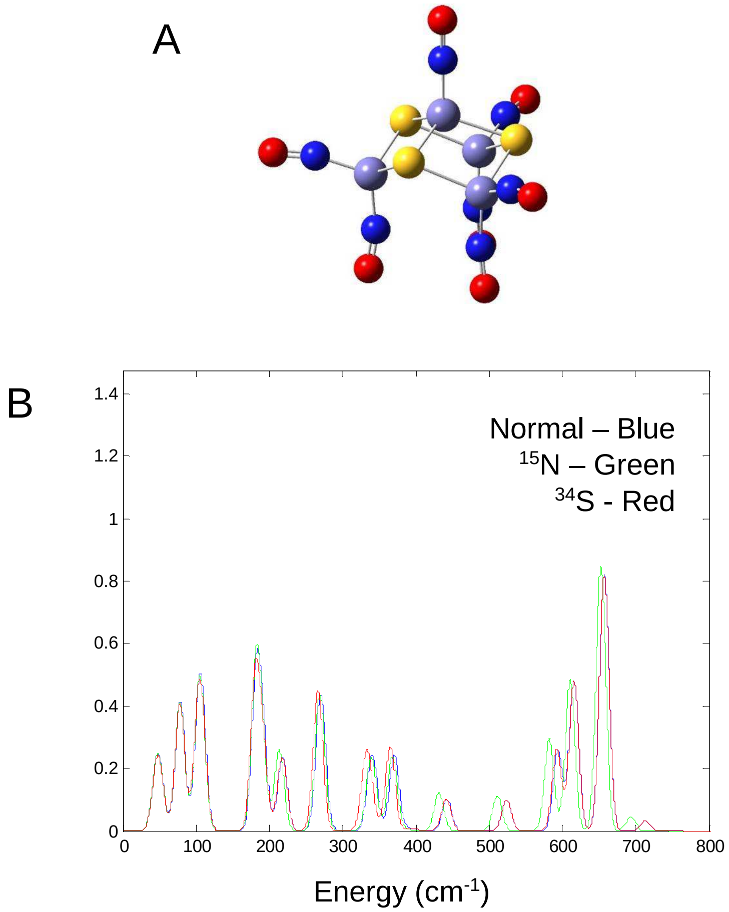

Figure S9. **DFT calculated NRVS spectra of RBS.** A) Structure of RBS anion, $[Fe_4(\mu_3\text{-}S)_3(NO)_7]^-$ (C_{3V}). Iron atoms are in light blue, sulfur in yellow, nitrogen in dark blue, oxygen in red. B) DFT calculated NRVS spectra of RBS, ^{15}NO-subsituted RBS and ^{34}S-substituted RBS, as indicated. The simulation shows good agreement with the previously reported ^{57}Fe PVDOS of $(Et_4N)[Fe_4(\mu_3\text{-}S)_3(NO)_7]$,[16] particularly in terms of the pattern of bands and their relative intensities right across the spectrum. The broad feature up to 100 cm^{-1} consists mainly of Fe-N=O bending modes with some S-Fe-S twisting. A sharp feature at ca. 110 cm^{-1} results from Fe-S-Fe scissoring. The slightly asymmetric band at 180 cm^{-1} is due to two vibrations, which are predominantly S-Fe-S scissoring/rocking motions. The band at ca. 280 cm^{-1} results from a combination of N-Fe-N twisting and S-Fe-S stretches, while those at ca. 350 cm^{-1} and 360 cm^{-1} are due predominantly to S-Fe-S stretching and rocking. Agreement between the calculated and experimental bands in the higher energy part of the spectrum (above 500 cm^{-1}) is less good, with the simulation approx. 50-60 cm^{-1} too high, but the overall pattern remains very well reproduced, with three clear bands. The first (ca. 595 cm^{-1} in the simulation) is due to N-Fe-N wagging with S-Fe stretching character. The second (ca. 620 cm^{-1}) is due to a combination of modes consisting of N-Fe-N symmetric stretches with various S-Fe-S vibrations. The third (ca. 660 cm^{-1}) is the N-Fe-N asymmetric equivalent and includes motion of the single Fe-N=O group in the center. Calculations were also performed with ^{15}N in place of ^{14}N, and ^{34}S in place of sulfide ^{32}S (with thiolate sulfur remaining as ^{32}S).

.

***Table S3*. DFT calculated vibrational modes and frequencies for RBS anion, [Fe$_4$(μ$_3$-S)$_3$(NO)$_7$]$^-$.** The highlight colors indicate vibrations that are: strong in NRVS and Raman (green); strong in NRVS and IR (red); strong in NRVS, Raman and IR (blue).

Mode	DFT Frequency (cm^{-1})
O-N-Fe-N-O Wag	77.04
O-N-Fe Bends/Twists/Rocks (**DD**)	79.13
N-Fe-N Wag / S-Fe-S Twists/Wag (**DD**)	103.65
Fe-S-Fe Scissor	108.85
S-Fe-S Scissor/Rock (**DD**)	182.55
N-Fe-N Rock / O-N-Fe Bend / Fe-S-Fe/S-Fe-S Scissor	192.05
N-Fe-N Rock / O-N-Fe Bend / Fe-Fe Stretch	216.47
N-Fe-N Rock / O-N-Fe Bend (**DD**)	219.40
N-Fe-N Twists / S-Fe-S Stretches (**DD**)	269.75
N-Fe-N Twist / O-N-Fe Bend / S-Fe-S Rock/Asymmetric Stretch	343.52
N-Fe-N Wag / O-N-Fe Bend / S-Fe-S Rock/Asymmetric Stretch (**DD**)	371.00
N-Fe-N Wag / O-N-Fe Bend / Fe-S-Fe Asymmetric Stretch	524.15
N-Fe-N Wags / O-N-Fe (Single) Bend / Fe-S-Fe Scissor/Stretch (**DD**)	594.02
N-Fe-N Symmetric Stretch / Fe-S-Fe Scissor/Wag	615.58
N-Fe-N Symmetric Stretch / Fe-S-Fe Scissor/Wag/Rock (**DD**)	616.30
N-Fe-N Asymmertric Stretches / N-Fe (Single) Stretch	656.59
N-Fe-N Asymmertric Stretches (**DD**)	656.59
N-Fe-N Asymmertric Stretches / N-Fe (Single) Stretch	659.56

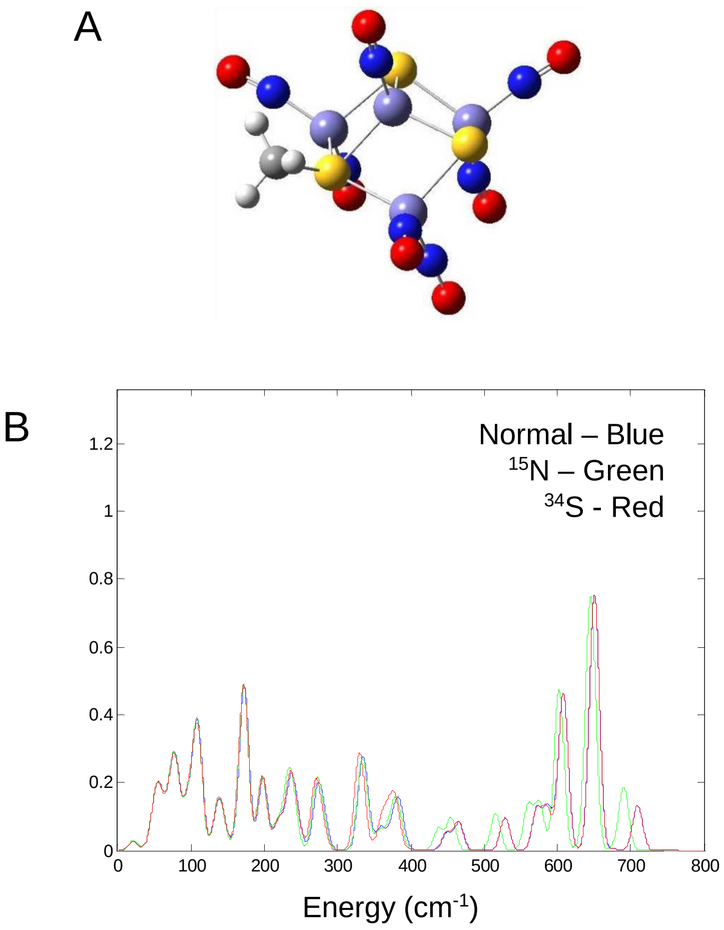

***Figure S10*. DFT calculated NRVS spectra of RBE with one thiolate bridge.** A) Structure of RBE with one thiolate bridge, $[Fe_4(\mu\text{-}S)_2(\mu\text{-}SCH_3)(NO)_7]$ (Cs). Iron atoms are in light blue, sulfur in yellow, nitrogen in dark blue, oxygen in red, methyl groups in gray. B) DFT calculated NRVS spectra of RBE with one thiolate bridge, along with ^{15}NO-subsituted and ^{34}S (sulfide)-substituted versions, as indicated. The mono-thiol substituted complex is of lower symmetry than RBS, resulting in a lifting of degeneracy of vibrational energies, with a consequent increase in the number of bands. Relative to the RBS simulation, the pattern of bands in the N-Fe-N region of the spectrum is slightly modified, with a predicted splitting of the band due to N-Fe-N wagging (at ca. 595 cm^{-1} in the RBS simulation).

***Table S4*. DFT calculated vibrational modes and frequencies for RBE with one thiolate bridge, $[Fe_4(\mu\text{-}S)_2(\mu\text{-}SCH_3)(NO)_7]$.** The highlight colors indicate vibrations that are: strong in NRVS and Raman (green); strong in NRVS and IR (red); strong in NRVS, Raman and IR (blue).

Mode	DFT Frequency (cm^{-1})
Fe-N=O Bend / Fe-S-Fe Twists/ Rocks	80.05
S-Fe-S Rock / Fe-S-Me Scissor	94.86
Fe-S-Fe Scissor	110.46
S-Fe-S Wag / Fe-S-Me Bend	136.39
Fe-N=O Bends / Fe-S-Fe Scissor/Rock	172.74
N-Fe-N Rock / Fe-N=O Bend / Fe-S-Fe Scissor / Fe-S-Me Scissor	198.35
N-Fe-N Rock / Fe-N=O Bend / Fe-S-Me Scissor	219.64
Fe-N=O Bend / N-Fe-N Rock / S-Fe-S Twist/Stretch	237.30
N-Fe-N Twist/Rock / S-Fe-S Stretches / S-Fe-S Twist / Fe-N=O Bend	273.10
N-Fe-N Rock / S-Fe-S Wag/Stretch / Fe-N=O Bend	282.48
S(Cys)-Fe(Central) Stretch / S(Bridging)-Fe(Non-Central) Stretch / S-Fe-S Wag	332.56
S-Fe-S Rock / S-Fe-S Asymmetric Stretch	335.57
N-Fe-N Twist / S-Fe-S Asymmetric Stretch	359.42
S-Fe-S Scissor/Stretch / Fe-N=O Bend (Single)	374.62
Fe-N=O Bend / N-Fe-N Twist / S-Fe-S Asymmetric Stretch/ Scissor	383.74
N-Fe-N Wag / S-Fe-S Rock / Fe-N=O Bend (Single)	449.48
N-Fe-N Wag / Fe-N=O (Single) Bend / S-Fe-S Scissor	465.91
N-Fe-N Wag / Fe-S-Fe Rock	528.72
Fe-N=O Bend (All) / N-Fe-N Wag / Fe-S-Fe Symmetric Stretch	572.48
Fe-N=O Bend (All) / N-Fe-N Wag / Fe-S-Fe Scissor	587.60
N-Fe-N Symmetric Stretch (Opposite S-Me)	606.00
N-Fe-N Symmetric Stretches (Adjacent to S-Me)	609.09
N-Fe-N Symmetric Stretches / Fe-S-Fe Rock (Adjacent to S-Me)	609.88
N-Fe-N Asymmetric Stretch / Fe-S-Fe Scissor / Fe-N Stretch (Single)	647.51
N-Fe-N Asymmetric Stretch / Fe-S-Fe Wag/Rock / Fe-N Stretch (Single)	649.62
N-Fe-N Asymmetric Stretch / Fe-S-Fe Wag / Fe-N Stretch (Single)	652.28
N-Fe-N Asymmetric Stretch / Fe-S-Fe Twist	652.76

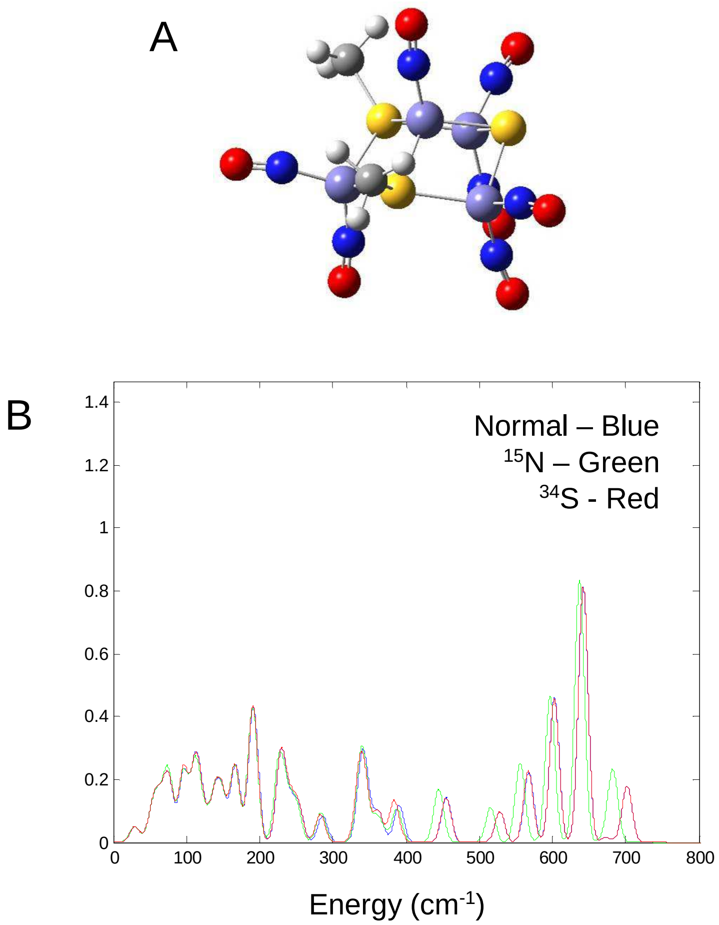

***Figure S11*. DFT calculated NRVS spectra of RBE with two thiolate bridges.** A) Structure of RBE with two thiolate bridges, $[Fe_4(\mu\text{-}S)(\mu\text{-}SCH_3)_2(NO)_7]^+$ (Cs). Iron atoms are in light blue, sulfur in yellow, nitrogen in dark blue, oxygen in red, methyl groups in gray. B) DFT calculated NRVS spectra of RBE with two thiolate bridges, along with ^{15}NO-subsituted and ^{34}S (sulfide)-substituted versions, as indicated. The di-thiol substituted complex is of lower symmetry than RBS, resulting in a lifting of degeneracy of vibrational energies, with a consequent increase in the number of bands. Relative to the RBS simulation, the pattern of bands in the N-Fe-N region of the spectrum is modified, with a significant splitting between the N-Fe-N wagging and N-Fe-N symmetric stretching bands.

***Table S5*. DFT calculated vibrational modes and frequencies for RBE with two thiolate bridges, $[Fe_4(\mu\text{-}S)(\mu\text{-}SCH_3)_2(NO)_7]^+$.** The highlight colors indicate vibrations that are: strong in NRVS and Raman (green); strong in NRVS and IR (red); strong in NRVS, Raman and IR (blue).

Mode	DFT Frequency (cm^{-1})
O=N-Fe-N=O Wag / S-Fe-S/Fe-S-Fe Rock	70.05
S-Fe-S Twist	76.65
Fe-S-Fe Rock	96.73
Fe-S-Fe Twist	111.16
Fe-Fe-S Rock	116.99
S-Fe-S Twist	138.78
Fe-Fe-S Scissor / Me Wagging	143.65
S-Fe-S/Fe-S-Fe Rock / Me Rocking	150.66
Fe-S-Fe Scissor /S-Fe-S Rock	167.16
Fe-S-Fe Scissor /S-Fe-S Rock	189.32
S-Fe-S Scissor	193.09
N-Fe-N Rock / S-Fe-S Wag	227.08
O=N-Fe Bend / N-Fe-N Rock / S-Fe-S Asymmetric Stretch	230.29
O=N-Fe Bend / N-Fe-N Rock / Fe-S-Fe Scissor	241.34
O=N-Fe Bend / N-Fe-N Rock / S-Fe-S Twist	246.46
N-Fe-N Rock / S-Fe-S Wag / Fe-N=O Bend	286.19
S-Fe-S Symmetric Stretch	336.30
S-Fe-S Asymmetric Stretches / Rock	340.67
S-Fe-S Asymmetric Stretch/Rock / Fe-N=O Bend (Single)	346.28
N-Fe-N Twist / S-Fe-S Stretches	360.16
Fe-N=O Bend (All) / N-Fe-N Wag / Fe-S(Bridging) Stretch	390.69
Fe-N=O Bend (Single) / Fe-S(Bridging) Stretch	453.03
Fe-N=O Bend (Single)	457.03
Fe-N=O Bend / Ne-Fe-N Wag	528.30
Fe-N=O Bend / Ne-Fe-N Wag	566.23
Fe-N=O Bend / Ne-Fe-N Wag	569.43
N-Fe-N Symmetric Stretches / Fe-S-Fe Rock	602.40
N-Fe-N Symmetric Stretch	602.77
N-Fe-N Symmetric Stretches / Fe-S-Fe Wag	603.14
N-Fe-N Asymmetric Stretches / Fe-S-Fe Rock	640.82
N-Fe-N Asymmetric Stretches / Fe-S-Fe Scissor	640.97
Fe-N Stretch (Single) / N-Fe-N Asymmetric Stretch	642.58
N-Fe-N Asymmetric Stretch / Fe-S-Fe Rock	644.08
Fe-N=O Bend / N-Fe-N Scissor	699.04
Fe-N=O Bend / N-Fe-N Scissor	699.47
Fe-N=O Bend / N-Fe-N Scissor	704.17

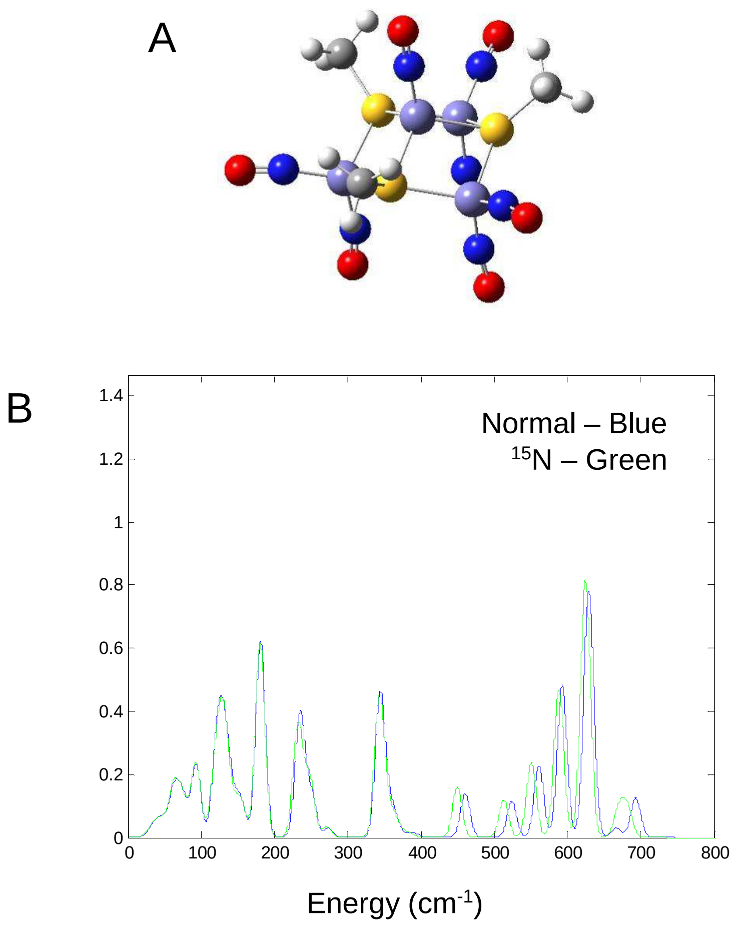

***Figure S12*. DFT calculated NRVS spectra of RBE with three thiolate bridges.** A) Structure of RBE with three thiolate bridges, $[Fe_4(\mu\text{-}SCH_3)_3(NO)_7]^{2+}$ (C_{3v}). Iron atoms are in light blue, sulfur in yellow, nitrogen in dark blue, oxygen in red, methyl groups in gray. B) DFT calculated NRVS spectra of RBE with three thiolate bridges along with a ^{15}NO-subsituted version, as indicated. Note that vibrations up to ~150 cm^{-1} are predominantly Me motions (more so than in other RBEs), and the NRVS spectrum seems to be a result of "recoils" rather than full vibrations. Relative to the RBS simulation, the pattern of bands in the N-Fe-N region of the spectrum is modified, with a significant splitting between the N-Fe-N wagging and N-Fe-N symmetric stretching bands.

***Table S6*. DFT calculated vibrational modes and frequencies for RBE with three thiolate bridges, $[Fe_4(\mu\text{-}SCH_3)_3(NO)_7]^{2+}$.** The highlight colors indicate vibrations that are: strong in NRVS and Raman (green); strong in NRVS and IR (red); strong in NRVS, Raman and IR (blue).

Mode	DFT Frequency (cm^{-1})
Me-S-Fe(Central) Scissor (Slight) / Me Motion	91.99
Fe-S-Fe Rock/Scissor (Slight) / Me Motion	93.57
Fe-S-Fe Rock/Scissor (Slight) / Me Motion	94.33
Fe-S-Fe Scissor (Slight) / Me Motion	120.41
Fe-S-Fe Scissor / S-Fe-S Wag (Slight) / Me Motion	121.47
S-Fe-S Twist (Slight) / Me Motion	127.15
S-Fe-S Rock/Scissor (Slight) / Me Motion	130.28
Fe-S-Fe Scissor (Slight) / Me Motion	131.07
Fe-S-Fe Twist (Slight) / Me Motion	135.02
Fe-S-Fe Twist (Slight) / Me Motion	141.64
S-Fe-S Rock / Me Motion	154.99
Fe-S-Fe Scissor/Rock / Fe-N=O Bends	180.71
S-Fe-S Rock / Fe-S-Fe Scissor	181.42
S-Fe-S Rock / Fe-S-Fe Scissor	181.93
N-Fe-N Rock / S-Fe-S Wag	231.41
N-Fe-N Rock / Fe-N=O Bends / Fe-S-Fe Stretches	235.55
N-Fe-N Rock / Fe-N=O Bends / Fe-S-Fe Stretches	237.25
Fe-N=O Bend (Single) / S-Fe-S Scissors/Stretches/Rock (DD)	344.41
S-Fe-S Asymmetric Stretches / Rock	353.31
Fe-N=O Bend (Single)	459.81
Fe-N=O Bend (Single)	461.81
N-Fe-N Wag / Fe-S-Fe Asymmetric stretches	524.33
N-Fe-N Wags (DD)	561.27
N-Fe-N Symmetric Stretches (DD) / Fe-S-Fe Twist	592.53
N-Fe-N Symmetric Stretches (DD) / Fe-S-Fe Wag	594.41
N-Fe-N Asymmetric Stretches (DD) / Fe-S-Fe Wag	627.28
N-Fe-N Asymmetric Stretches / Fe-S-Fe Rock	628.42
N-Fe-N Asymmetric Stretches / Fe-S-Fe Scissor	629.02
Fe-N Stretch (Single)	633.11

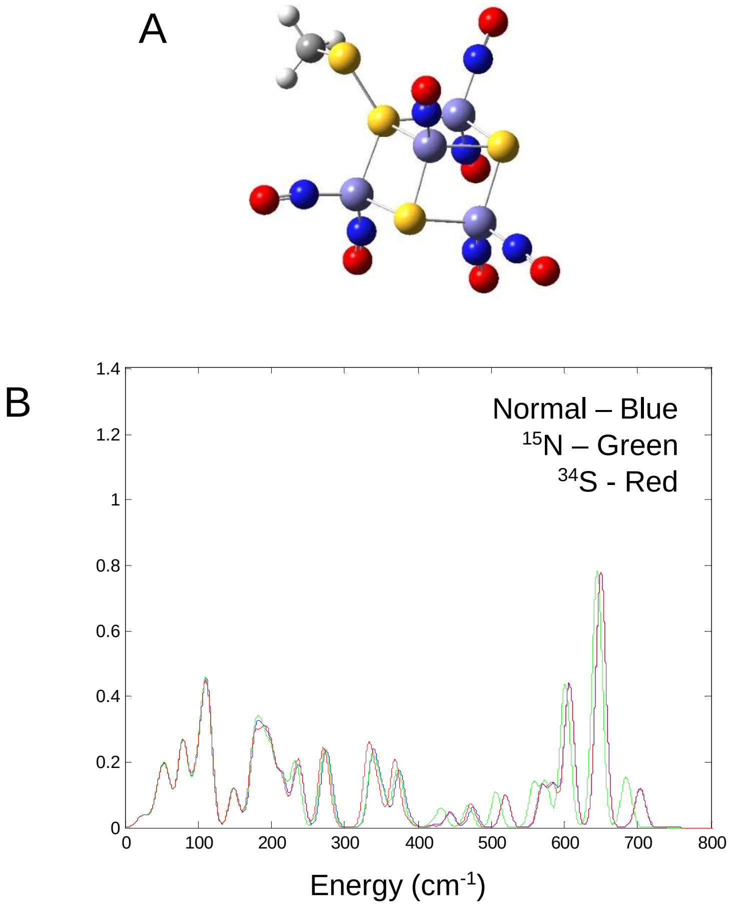

***Figure S13*. DFT calculated NRVS spectra of RBE with one persulfide thiolate bridge.** A) Structure of RBE with one persulfide thiolate bridge, $[Fe_4(\mu\text{-}S)_2(\mu\text{-}SSCH_3)(NO)_7]$ (Cs). Iron atoms are in light blue, sulfur in yellow, nitrogen in dark blue, oxygen in red, methyl groups in gray. B) DFT calculated NRVS spectra of RBE with one persulfide thiolate bridge, along with ^{15}NO-subsituted and ^{34}S (sulfide)-substituted versions, as indicated.

Table S7. **DFT calculated vibrational modes and frequencies for RBE with one persulfide thiolate bridge, $[Fe_4(\mu\text{-}S)_2(\mu\text{-}SSCH_3)(NO)_7]$.** The highlight colors indicate vibrations that are: strong in NRVS and Raman (green); strong in NRVS and IR (red); strong in NRVS, Raman and IR (blue).

Mode	DFT Frequency (cm^{-1})
Fe-S-Fe Twist	76.78
N-Fe-N Rock / Fe-S-Fe Wag	82.66
Fe-S-S(Me) Wag /S-Fe-S Rock	97.77
S-Fe-S Twist / Fe-S-S(Me) Scissor	106.73
Fe-S-Fe Scissor	111.09
Fe-S-Fe Rock	116.37
S-Fe-S Twist/Wag / S-S-Me Scissor	148.91
S-Fe-S Scissor/Rock	177.78
Fe-N=O Bend / N-Fe-N Rock / Fe-S-Fe Scissor /Wag / S-S-Me Scissor	189.09
Fe-N=O Bend / N-Fe-N Rock / Fe-S-Fe Scissor / Fe-S-S(Me) Scissor	200.54
Fe-N=O Bend / N-Fe-N Rock /S-Fe-S Scissor/Wag / S-S-Me Scissor	215.04
Fe-N=O Bend / N-Fe-N Rock / Fe-S-Fe Twist	231.37
Fe-N=O Bend / N-Fe-N Rock / S-Fe-S Wag / S-S-Me Scissor	238.67
Fe-N=O Bend / N-Fe-N Twist / S-Fe-S Stretches/Scissors	273.20
Fe-N=O Bend / N-Fe-N Twists/Rock / S-Fe-S Symmetric Stretch/Scissors	281.36
S-Fe-S Stretches/Scissors/Rocks / S-S-Me Scissor	338.05
S-Fe-S Stretches/Scissors/Rocks / S-S-Me Scissor	339.58
S-Fe-S Symmetric Stretches/Scissors / S-S-Me Scissor	349.67
N-Fe-N Twist / N-Fe-S(-S-Me) Twist	354.51
Fe-N=O Bends / S-Fe-S Scissor/Stretches	373.45
N-Fe-N Twist / S-Fe-S Scissor/Stretches/Rocks	375.62
Fe-N=O Bend (Single) / N-Fe-N Wag / S-Fe-S Rock	444.45
Fe-N=O Bend (Single) / N-Fe-N Wag / Fe-S-S Asymmetric Stretch	475.86
Fe-N=O Bends / N-Fe-N Wags / Fe-S-Fe Twist	519.77
Fe-N=O Bends (All) / N-Fe-N Wags / Fe-S-Fe Stretches/Scissors	570.32
Fe-N=O Bends (All) / N-Fe-N Wags / Fe-S-Fe Stretches/Scissors	585.96
N-Fe-N Symmetric Stretches	605.08
N-Fe-N Symmetric Stretches	605.42
N-Fe-N Symmetric Stretch	609.55
Fe-N Stretch (Single) / Fe-S-Fe Rock	646.45
Fe-N Stretch (All) / Fe-S-Fe Twist	649.18
N-Fe-N Asymmetric Stretch	651.62
Fe-N Stretch (All) / Fe-S-Fe Scissor	651.84
Fe-N=O Bends / N-Fe-N Scissors	700.54
Fe-N=O Bends / N-Fe-N Scissors	703.60
Fe-N=O Bends / N-Fe-N Scissors	707.65

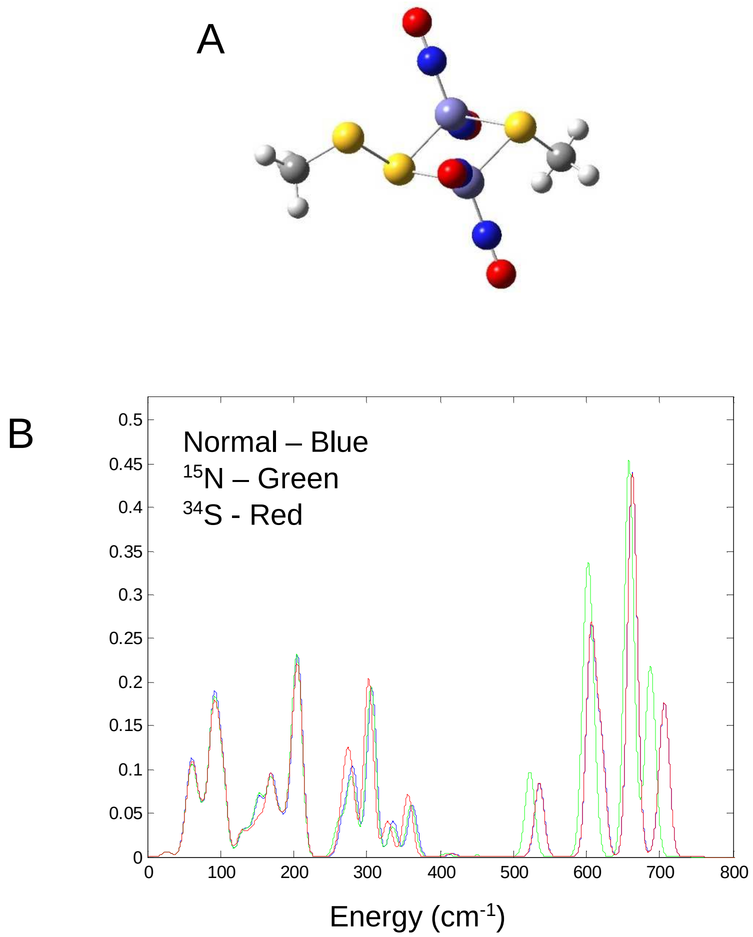

***Figure S14*. DFT calculated NRVS spectra of RRE with one persulfide bridge.** A) Structure of RRE with one persulfide bridge, $[Fe_2(\mu\text{-}SCH_3)(\mu\text{-}SSCH_3)(NO)_4]$ (Cs). Iron atoms are in light blue, sulfur in yellow, nitrogen in dark blue, oxygen in red, methyl groups in gray. B) DFT calculated NRVS spectra of RRE with one persulfide bridge, along with ^{15}NO-subsituted and ^{34}S (sulfide)-substituted versions, as indicated.

***Table S8*. DFT calculated vibrational modes and frequencies for RRE with one persulfide bridge, [Fe_2(μ-SCH_3)(μ-$SSCH_3$)$(NO)_4$].** The highlight colors indicate vibrations that are: strong in NRVS and Raman (green); strong in NRVS and IR (red).

Mode	DFT Frequency (cm^{-1})
O=N-Fe-N=O Scissor	59.43
O=N-Fe-N=O Wag / Me-S-S Rock	59.87
O=N-Fe-N=O Scissor	65.67
N-Fe-S Scissor (Slight)	77.29
Fe-S-Fe Twist	91.17
S-S-S Wag / Fe-S-Fe Rock (Slight)	102.75
S-S-Me Wag / Fe-S-Fe Rock (Slight)	153.42
S-Fe-S Scissor	169.50
S-S-Me Wag / Fe-S-Fe Rock (Slight)	183.31
Fe-S-Fe Wag (Slight) / S-S-Me Scissor	199.10
Fe-S-Fe Scissor / S-S-Me Scissor	206.65
Fe-N=O Bend/ N-Fe-N Rock / Fe-S-Fe Twist	268.19
Fe-N=O Bend / N-Fe-N Rock / S-Fe-S Wag	280.91
S-Fe-S Scissor/Stretch	281.57
N-Fe-N Wag / Fe-S-Fe Asymmetric Stretch	306.76
S-Fe-S Scissor/Stretch / S-S-Me Scissor	336.09
S-Fe-S Stretch/Rock / S-S-Me Scissor	362.64
Fe-N=O Bend / N-Fe-N Wag / Fe-S-Fe Rock	535.97
N-Fe-N Asymmetric Stretch / Fe-S-Fe Rock	605.37
N-Fe-N Symmetric Stretch / Fe-S-Fe Scissor	607.29
Fe-N=O Bend / N-Fe-N Wag / Fe-S-Fe Symmetric Stretch	620.60
N-Fe-N Asymmetric Stretch / Fe-S-Fe Twist	661.80
N-Fe-N Asymmetric Stretch / Fe-S-Fe Wag	663.67
Fe-N=O Bend / N-Fe-N Scissor / Fe-S-Fe Rock	701.65
Fe-N=O Bend / N-Fe-N Scissor / Fe-S-Fe Scissor	708.00

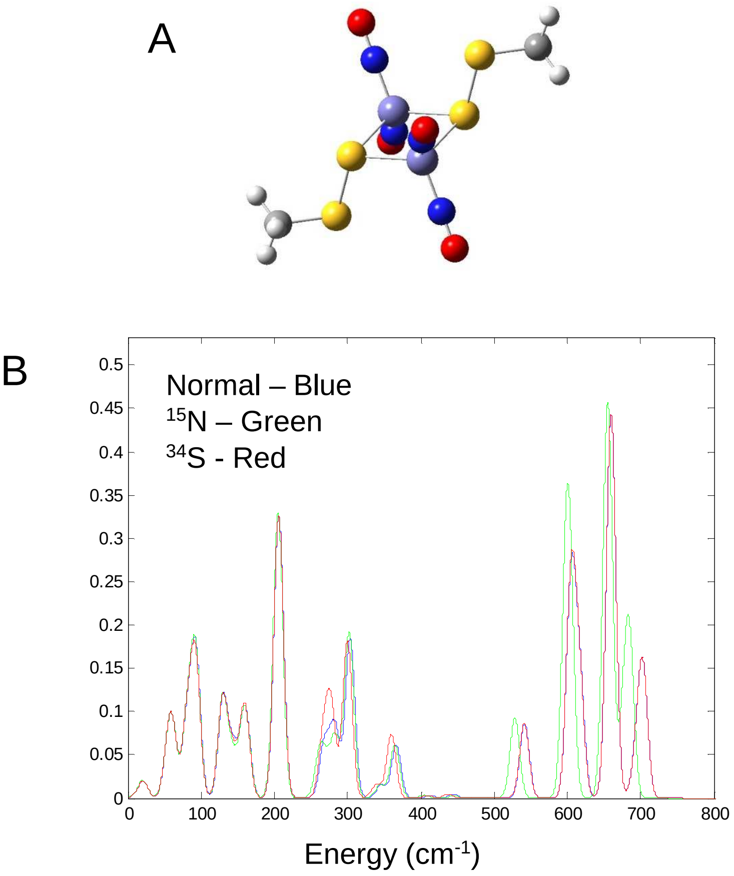

***Figure S15*. DFT calculated NRVS spectra of RRE with two persulfide bridges.** A) Structure of RRE with two persulfide bridges, $[Fe_2(\mu\text{-}SSCH_3)_2(NO)_4]$ (C_{2h}). Iron atoms are in light blue, sulfur in yellow, nitrogen in dark blue, oxygen in red, methyl groups in gray. B) DFT calculated NRVS spectra of RRE with two persulfide bridges, along with ^{15}NO-subsituted and ^{34}S (sulfide)-substituted versions, as indicated.

***Table S9*. DFT calculated vibrational modes and frequencies for RRE with two persulfide bridges, $[Fe_2(\mu\text{-}SSCH_3)_2(NO)_4]$.** The highlight colors indicate vibrations that are: strong in NRVS and Raman (green); strong in NRVS and IR (red).

Mode	DFT Frequency (cm^{-1})
O=N-Fe-N=O Rock / S-S-Me Rock	53.56
O=N-Fe-N=O Wag / S-S-Me Rock	60.22
O=N-Fe-N=O Scissor / Fe-S-Fe Rock / S(Cys)-S-S Wag	81.09
Fe-S-Fe Twist	92.78
S-Fe-S Rock	129.99
Fe-N=O Bend / N-Fe-N Rock / S-Fe-S Wag / S-S-Me Rock	143.20
Fe-S-Fe Rock (Slight)	157.80
Fe-S-Fe Scissor	162.50
S-Fe-S Scissor/Stretch (Slight) / S-S-Me Scissor	204.07
S-Fe-S Scissor / S-S-Me Scissor	206.95
Fe-N=O Bend / N-Fe-N Rock / S-Fe-S Asymmetric Stretch	267.82
Fe-N=O Bend / N-Fe-N Rock/ S-Fe-S Rock	277.38
N-Fe-N Twist / S-Fe-S Scissor/Stretch	283.75
Fe-N=O Bend / N-Fe-N Rock/Twist / S-Fe-S Asymmetric Stretch	303.60
N-Fe-N Wag / S-Fe-S Rock/Asymmetric Stretch	366.58
Fe-N=O Bend / N-Fe-N Wag / Fe-S-Fe Rock	541.80
N-Fe-N Asymmetric Stretch	604.18
N-Fe-N Symmetric Stretch / Fe-S-Fe Scissor	606.42
Fe-N=O Bend / N-Fe-N Wag / Fe-S-Fe Symmetric Stretch / S-Fe-S Rock	617.14
N-Fe-N Asymmetric Stretch / Fe-S-Fe Twist	658.88
N-Fe-N Asymmetric Stretch / Fe-S-Fe Wag	660.75
Fe-N=O Bend / N-Fe-N Scissor / Fe-S-Fe Rock	695.06
Fe-N=O Bend / N-Fe-N Scissor / Fe-S-Fe Scissor	703.70

Supporting References

[1] a) N. P. Tucker, M. G. Hicks, T. A. Clarke, J. C. Crack, G. Chandra, N. E. Le Brun, R. Dixon, M. I. Hutchings, *Plos One* **2008**, *3*; b) J. C. Crack, J. Green, A. J. Thomson, N. E. Le Brun, In *Metallproteins*, J. C. Fontecilla-Camps, Y. Nicolet, Eds, Humana Press, **2014**, *Vol. 1122*, p33-48; c) J. C. Crack, E. L. Dodd, F. Knowles, M. M. Al Bassam, S. Kamali, A. A. Holland, S. P. Cramer, C. J. Hamilton, M. K. Johnson, A. J. Thomson, M. I. Hutchings,N. E. Le Brun, *J. Biol. Chem.* **2015**, *290*, 12689- 12704.

[2] B. Zhang, J. C. Crack, S. Subramanian, J. Green, A. J. Thomson, N. E. Le Brun, M. K. Johnson, *Proc. Nat. Acad. Sci. U. S. A.* **2012**, *109*, 15734-15739.

[3] J. C. Crack, C. D. den Hengst, P. Jakimowicz, S. Subramanian, M. K. Johnson, M. J. Buttner, A. J. Thomson, N. E. Le Brun, *Biochemistry* **2009**, *48*, 12252-12264.

[4] M. C. Kennedy, Emptage, M. H., Beinert, H., *J. Biol. Chem.* **1984**, *259*, 3145-3151.

[5] a) M. C. Smith, Y. Xiao, H. Wang, S. J. George, D. Coucovanis, M. Koutmos, W. Sturhahn, E. E. Alp, J. Zhao, S. P. Cramer, *Inorg. Chem.* **2005**, *44*, 5562-5570; b) Y. Xiao, K. Fisher, M. C. Smith, W. Newton, D. A. Case, S. J. George, H. Wang, W. Sturhahn, E. E. Alp, J. Zhao, Y. Yoda, S. P. Cramer, *J. Am. Chem. Soc.* **2006**, *128*, 7608-7612.

[6] a) S. Kamali, H. Wang, D. Mitra, H. Ogata, W. Lubitz, B. C. Manor, T. B. Rauchfuss, D. Byrne, V. Bonnefoy, F. E. Jenney Jr., M. W. W. Adams, Y. Yoda, E. Alp, J. Zhao, S. P. Cramer, *Angew. Chemie Int. Ed.* **2013**, *52*, 724 –728; b) L. Lauterbach, H. Wang, M. Horch, L. B. Gee, Y. Yoda, Y. Tanaka, I. Zebger, O. Lenz, S. P. Cramer, *Chem. Sci.* **2015**, *6*, 1055-1060; c) H. Wang, E. Alp, Y. Yoda, S. Cramer, in *Metalloproteins*, (Eds.: J. C. Fontecilla-Camps, Y. Nicolet), Humana Press, **2014**, *Vol. 1122*, p125-137; d) S. P. Cramer, Y. Xiao, H. Wang, Y. Guo, M. C. Smith, *Hyperfine Int.* **2006**, *170*, 47-54; e) H. Ogata, T. Kramer, H. Wang, D. Schilter, V. Pelmenschikov, M. van Gastel, F. Neese, T. B. Rauchfuss, L. B. Gee, A. D. Scott, Y. Yoda, Y. Tanaka, W. Lubitz, S. P. Cramer, *Nat. Commun.* **2015**, *6*, 7890; f) H. Wang, Y. Yoda, S. Kamali, Z.-H. Zhou, S. P. Cramer, *J. Synch. Rad.* **2012**, *19*, 257-263.

[7] W. Sturhahn, *Hyperfine Int.* **2000**, *125*, 149-172.

[8] W. Dong, P. He, J. Wang, Z. Zhou, H. Wang, *Infrared Phys.Tech.* **2013**, *56*, 51-56.

[9] K. Lagrec, D. C. Rancourt, 1.0 ed., Department of Physics, University of Ottawa, Canada, **1998**.

[10] J. P. Perdew, J. A. Chevary, S. H. Vosko, K. A. Jackson, M. R. Pederson, D. J. Singh, C. Fiolhais, *Phys. Rev. B* **1992**, *46*, 6671-6687.

[11] D. Mitra, V. Pelmenschikov, Y. Guo, D. A. Case, H. Wang, W. Dong, M.-L. Tan, T. Ichiye, J. Francis E. Jenney, M. W. W. Adams, Y. Yoda, J. Zhao, S. P. Cramer, *Biochemistry* **2011**, *50*, 5220–5235.

[12] a) B. M. Leu, M. Z. Zgierski, G. R. A. Wyllie, W. R. Scheidt, W. Sturhahn, E. E. Alp, S. M. Durbin, J. T. Sage, *J. Am. Chem. Soc.* **2004**, *126*, 4211-4227; b) W. Sturhahn, T. S. Toellner, E. E. Alp, X. Zhang, M. Ando, Y. Yoda, S. Kikuta, M. Seto, C. W. Kimball, B. Dabrowski, *Phys. Rev. Lett.* **1995**, *74*, 3832-3835; c) R. Rüffer, A. I. Chumakov, *Hyperfine Int.* **1996**, *97-8*, 589-604; d) A. Q. R. Baron, A. I. Chumakov, R. Rüffer, H. Grünsteudel, H. F. Grünsteudel, O. Leupold, *Europhys. Lett.* **1996**, *34*, 331-336; e) V. S. Oganesyan, J. E. Barclay, S. M. Hardy, D. J. Evans, C. J. Pickett, U. A. Jayasooriya, *Chem. Commun.* **2003**, 214-215; f) M. Seto, Y. Yoda, S. Kikuta, X. W. Zhang, M. Ando, *Phys. Rev. Lett.* **1995**, *74*, 3828-3831; g) A. I. Chumakov, R. Ruffer, H. Grunsteudel, H. F. Grunsteudel, G. Grubel, J. Metge, O. Leupold, H. A. Goodwin, *Europhys. Lett.* **1995**, *30*, 427-432; h) U. A. Jayasooriya, J. N. T. Peck, J. E. Barclay, S. M. Hardy, A. I. Chumakov, D. J. Evans, C. J. Pickett, V. S. Oganesyan, *Chem. Phys. Lett.* **2011**, *518*, 119-123; i) U. A. Jayasooriya, S. A. Malone, A. I. Chumakov, R. Rüffer, A. R. Overweg, C. R. Nicklin, *ChemPhysChem* **2001**, *2*, 177-180.

[13] Y. Xiao, H. Wang, S. J. George, M. C. Smith, M. W. W. Adams, J. Francis E. Jenney, W. Sturhahn, E. E. Alp, J. Zhao, Y. Yoda, A. Dey, E. I. Solomon, S. P. Cramer, *J. Am. Chem. Soc.* **2005**, *127*, 14596-14606.

[14] T. Petrenko, S. D. George, N. Aliaga-Alcalde, E. Bill, B. Mienert, Y. Xiao, Y. Guo, W. Sturhahn, S. P. Cramer, K. Wieghardt, F. Neese, *J. Am. Chem. Soc.* **2007**, *129*, 11053-11060.

[15] a) R. J. Dai, Ke, S. C., *J. Phys. Chem. B* **2007**, *111*, 2335- 2346; b) S. F. Ye, F. Neese, *J. Am. Chem. Soc.* **2010**, *132*, 3646-3647.

[16] Z. J. Tonzetich, H. Wang, D. Mitra, C. E. Tinberg, L. H. Do, F. E. Jenney, Jr., M. W. W. Adams, S. P. Cramer, S. J. Lippard, *J. Am. Chem. Soc.* **2010**, *132*, 6914-6916.

[17] J. C. Crack, L. J. Smith, M. R. Stapleton, J. Peck, N. J. Watmough, M. J. Buttner, R. S. Buxton, J. Green, V. S. Oganesyan, A. J. Thomson, N. E. Le Brun, *J. Am. Chem. Soc.* **2011**, *133*, 1112-1121.
